# Is the Millennial Generation Left Behind? Inter-Cohort Labour Income Inequality in a Context of Economic Shock

**DOI:** 10.1007/s11205-022-02958-x

**Published:** 2022-06-22

**Authors:** Marta Escalonilla, Begoña Cueto, María José Pérez-Villadóniga

**Affiliations:** Applied Economics Department, University of Oviedo, Av. del Cristo, 33006 Oviedo, Asturias, Spain

**Keywords:** Income inequality, The great recession, Cohort analysis, Age-period-cohort modelling, Young generations, D31, E24, J31

## Abstract

This paper provides new evidence on how intergenerational income inequality evolves during the period 2005–2019. Using the Continuous Sample of Working Histories (CSWH), which includes administrative data about working lives and personal characteristics of Spanish workers, we shed light on the effect of the Great Recession on income inequality between cohorts in Spain. As a proxy of income, we employ monthly earnings data, provided by the CSWH. From a life course approach, we use two age-period-cohort (APC) models which allow us to separately identify three components: cohort, age and period effects. First, we examine *relative* earnings which will reveal whether there are income differences between generations. Second, we measure how *absolute* earnings have developed over time. Our results suggest that some generations are more disadvantaged in terms of income by their year of birth than others. Likewise, the evidence points out that the economic context experienced by a generation in their transition to the labour market is a key factor in the development of their income.

## Introduction

The literature on economic inequality is growing as a result of increasing interest in measuring and understanding the level, causes and development of income inequality. This extensive research emerging in recent years focuses on the evolution of income inequality over age (Almås et al., 2011; Hungerford, 2020) or time globally, such as Hammar and Waldenström (2020), and in specific countries, such as Blundell and Etheridge (2010), Fuchs-Schundeln et al. (2010), Domeij and Floden (2010), evidencing its potential effect on growth, social stability, and welfare.

During the last decade, several studies have examined the relationship between inequality and business cycles (Barlevy & Tsiddon, 2006), mainly as a consequence of the recent global recession and with the aim of understanding the probable distributional implications that it generated. The evidence found in the literature is mixed. Concretely, some authors find that income inequality follows a countercyclical pattern, that is, an increase of income inequality during an economic downturn and a decrease during an economic expansion (Hoynes et al., 2012; Guvenen et al., 2014; Hoover et al., 2009; Camacho & Palmieri, 2019). Other authors, instead, suggest the opposite. This is the case of Morin (2019) and Karonen and Niemelä (2020), who find that income inequality is procyclical.

Therefore, the evidence shows that changes in income inequality are associated with the business cycle. However, the evolution of income inequality at the aggregate level cannot reveal the complexities of income dynamics in terms of inequalities between cohorts. To do so, a life course perspective is required. Life course is defined as life trajectories in which income development varies by cohort (Elder, 1998; Ryder, 1965). The birth year places people in specific birth cohorts and, therefore, according to particular social changes. The impact of an event like an economic shock depends on when it affects in the life stage of the cohort (Elder, 1998). As Karonen and Niemelä (2020) state “this perspective emphasizes that certain cohorts could experience an accumulating effect due to an economic shock”.

In terms of income inequality, it raises questions like: Are younger generations better off than older ones? Are young and old generations becoming more unequal? What is the role played by a macroeconomic shock in shaping income inequality across cohorts over the life course? Thus, a cohort-based analysis over the life cycle may help us to better understand the drivers of inter-cohort inequality and the ways in which labour markets have changed during the last years. In consequence, it allows us to separately identify age, time, and generational (cohort) effects. In other words, it isolates the effect of cohort membership on income from the effect of general economic growth, which mostly increases incomes on a period-to-period basis and from the effect of age, which typically lets incomes peak around the midpoint of a working career.

The evidence using this perspective shows that there are significant generational differences in economic measures such as income, wealth and consumption (Chauvel and Schöder, 2014; Karonen & Niemelä, 2020; Berloffa & Villa, 2010; Lim & Zeng, 2016). Likewise, empirical evidence supports the concern that younger generations are less well off than members of earlier generations in terms of lower earnings and less wealth (Gale et al., 2020; Kurz et al., 2018).

In this context, the aim of this paper is to provide new evidence relative to how inter-cohort income inequality^1^ develops in Spain during the period 2005–2019, distinguishing three components: cohort, age and period effects. The period analysed, includes several phases of the economic cycle. Between 2005 and 2007, Spain enjoyed a phase of economic growth, followed by the Great Recession that started in 2008. Subsequently, from 2014 onwards a phase of economic expansion begins. Therefore, this study offers new evidence on the impact of the economic crisis on inter-generational income inequality.^2^

The evolution of income inequality in Spain has been characterized by being strongly countercyclical (Izquierdo & Lacuesta, 2012; Pijoan-Mas & Sánchez-Marcos, 2010; Simón, 2009). Income inequality declined substantially during the 1997–2007 expansion, and then rose again strongly during the Great Recession (Anghel et al., 2018; Bonhomme & Hospido, 2017). Additionally, it has been found that, particularly at the bottom of the distribution, earnings and income fell considerably during recessions and increased during economic booms (Anghel et al., 2018; Carrasco et al., 2015; Izquierdo & Lacuesta, 2012). However, the cohort dimension is not considered in any of these studies and its inclusion opens up a new perspective on the evolution of income inequality.

At a descriptive level, there is evidence that compares the working conditions experienced by the youngest cohorts in Spain with those of previous generations before and after the 2008 crisis (Hernández de Cos, 2019; Puente & Regil, 2020). In general terms, it is shown that, before the crisis, each new generation reached an annual income on average higher than that of the previous generation as the workers accumulated work experience. However, this changes with the onset of the recession. Specifically, it is observed that the annual income of the young generations has decreased compared to previous generations.

In this paper, we follow the most recent methodologies designed to analyse inter-cohort income dynamics and carry out our analysis from two approaches. First, we examine *relative* earnings which will reveal whether there are differences between generations in terms of earnings. Conditional on the possible existence of inter-cohort inequality, secondly, we measure how *absolute* earnings have developed over time. Thus, we will be able to discover for which cohorts the earnings development has stopped or slowed down. This perspective, hence, shows whether the economic crisis particularly affected the absolute income dynamics of some cohorts, but not that of other cohorts.

We contribute to the literature in several aspects. First, we provide new evidence relative to how inter-cohort income inequality develops in relative and absolute terms in a context of economic crisis, considering at the same time the three dimensions of cohort, period and age.

Second, this study uses administrative data, which contains information on working conditions as well as socioeconomic characteristics of individual workers. One of the advantages of this dataset is that it allows us to follow the labour market trajectories of each individual and, thus, of cohorts over time. Hence, unlike most of the previous literature, which relies on longitudinal single-cohort or cross-sectional designs and therefore cannot distinguish cohort effects, we have longitudinal data from multiple cohorts. More specifically, we focus on cohorts born between 1950 and 1994, who are working during 2005–2019. Hence, we provide new evidence relative to income inequality between three generations, such as the baby-boom generation (born between the late 1940s and early 1960s), the generation X (born between the mid-60s and the late 1970s) and the Millennial generation (born between the early 1980s and 1990s). Since the main objective of this paper is to answer the question whether younger generations are better off than older ones, the referent generation when we compare these three generations will be the Millennial generation.

Finally, we include in our analysis a gender perspective. Previous literature on inter-cohort inequality only examines men. Therefore, this approach will allow us to identify possible gender differences in the dynamics of income inequality between cohorts.

The remainder of the paper is organized as follows. In next section, we review the related literature. Section 3 outlines our empirical strategy for estimating the impact of the Great Recession on income inter-cohort inequality. Section 4 describes the data and sample. Section 5 presents the estimations results and discussion. Section 6 concludes, summarizing our main results.

## Literature Review

Despite the extensive literature on income inequality, little is known about the role of cohort membership because it is often excluded from the analysis. Hence, the relationship between income dynamics and cohorts is not enough discussed.

It is becoming increasingly clear that cohorts play a potential role in producing social change because social contexts and historical circumstances vary from cohort to cohort (Ryder, 1965). A cohort is defined as the aggregate of individuals who experienced the same event in the same time interval (Mannheim, 1928). As Ryder (1965) notes “if change does occur, it differentiates cohorts from one another”. The consequences of change may persist in the subsequent behavior of these individuals and thus of their cohorts. In line with this, Schuman and Scott (1989) find that events occurring during adolescence and young adulthood leave a deep-rooted mark, leading to cohort differences in beliefs about the importance of national and world events, and thus a cohort effect.

From an economic viewpoint, these discrepancies between cohorts could be considered inequalities because some cohorts may have a more or less favourable entry into the labour market due to the specific economic situation they face. Thus, an economic expansion or downturn may play a key role in how a cohort is able to establish itself during changing market situations. Malmendier and Nagel (2011) analyse whether economic shocks affect individual financial risk decisions. They find that cohorts that have become adults during economic booms are more likely to profit from that favourable market situation. Cohorts who are “scarred” by an economic downturn, instead, may be more risk‐averse and have a more disadvantaged economic trajectory from a life course perspective. This evidence leads to the idea that there are “lucky and less lucky generations”, in which the year of birth of the cohorts is key in social dynamics (Chauvel, 2013).

Research focusing on inter-cohort income inequality points out that the “baby boomer” generation has benefitted most from its birth cohort compared with other generations in terms of income (Chauvel & Schröder, 2014). Using a cohort analysis, they focus on cohorts born between 1935 and 1975 and the period 1985–2005. They find income differences between generations, being the baby boomer generation the luckiest. Such inequalities between generations are much stronger in conservative European welfare states, compared to social democratic and liberal welfare states.

Similar evidence is found by Chauvel and Schrörder (2015). They examine how belonging to a certain birth cohort influences disposable incomes in France, Germany and US and whether these countries advantage some birth cohorts in terms of income while disadvantaging others. In particular, the authors focus on birth cohorts 1920–1975 and the period 1985–2005. They find that cohorts born between 1940 and 1950 have disposable incomes well above what one would expect if all cohorts had equally participated in long-run increases in disposable incomes. Regarding France, the authors show more pronounced cohort differences compared to Germany and US. They argue that older generations have monopolized lucrative positions and social transfers, to the detriment of generations born after 1950. In the same way, Freedman (2017) analyses the variation in income generational inequalities across 8 different countries during the period 1985–2005. The evidence shows again that cohorts born after 1970 have experienced fewer earning opportunities, relative to cohorts born between 1950 and 1970.

However, these studies only observe differences between cohorts without emphasizing how the economic context that these cohorts experience affects intergenerational inequality. Some authors point out that inter-cohort income inequalities are greatly affected by macroeconomic shocks (Mayer, 2005). Cohorts entering the job market during times of austerity and economic downturn are in a more disadvantaged position, compared to cohorts entering during an economic growth, with regard to attaining similar career options. For instance, Berloffa and Villa (2010) explore the evolution of Italian household income over the period 1989–2004 and find that young generations face economic difficulties. In particular, while those born in the 1930s and 1940s gain about 8 percent over the preceding cohorts, the younger ones record an average loss of about 5 percent, which is a result of the economic situation, its adverse effects on younger workers and different socio‐political reforms.

In the research of Karonen and Niemelä (2020), the development of income distribution across periodic economic fluctuations in relation to cohorts and age groups is examined. Concretely, they analyse the Finnish case covering the period of 1966–2015. They find that the main effects on relative income are basically driven by period and cohort effects. This result suggests a link between the effects of economic shocks and cohort placement on labour market entry. Moreover, absolute income analysis suggests that an economic shock produce a stagnation in income development, which affects the younger cohorts more intensely.

## Methodology

In general terms, age–period–cohort models (henceforth APC) try to explain outcomes distinguishing the effect of three different influences: the individual’s age, linked to the life cycle (*α*_*a*_), birth cohort membership (*γ*_*c*_), and period of measurement (*π*_*p*_). Thus, the equation of APC models can be written as follows:

1 $$y^{apc} = \mu + \alpha_{a} + \pi_{p} + \gamma_{c} (APC)$$

However, all APC models are affected by an “identification problem” (Glenn, 1976), in which each component is a combination of the other two, that is, they are collinear. The literature has attempted to provide a solution to this problem (Yang & Land, 2013; Yang et al., 2008), but these approaches have limitations and disadvantages (Freedman, 2017; Pampel & Hunter, 2012).

In view of the limitations of these models, firstly, we use an APC-detrended (APCD) model, which was proposed by Chauvel (2012). Thus, we examine *relative* earnings which will reveal whether there are inequalities between generations in terms of earnings. This method differentiates between “linear trends” and fluctuations. More specifically, this approach displays how the effects of age, period and cohort on earnings fluctuate around a linear trend, which is equal to zero. In other words, this model is a “bump” detector that shows how earnings deviate from the general income trend through different cohorts, ages and years.^3^

The APCD model imposes two sets of constraints: the vectors of age, period and cohort parameters have a zero-sum and a zero-slope. Moreover, the first and the last cohorts of the estimations of the models are excluded to improve the confidence intervals of the parameters. This produces a unique estimate of detrended age–period–cohort effects and leads to solve the traditional identification problem of the APC model. The APCD model can be expressed as follows:

2 $$\begin{gathered} y^{apc} = \alpha_{a} + \pi_{p} + \gamma_{c} + \alpha_{{^{0} }} rescale(a) + \gamma_{0} rescale(c) + \beta_{0} + \sum {_{j} \beta_{j} X_{j} + \varepsilon_{i} } \hfill \\ \left\{ \begin{gathered} \sum {(\alpha_{a} )} = 0;\quad \sum {(\pi_{p} )} = 0;\quad \sum {(\gamma_{c} )} = 0 \hfill \\ Slope_{p} (\alpha_{a} ) = 0;\quad Slope_{a} (\pi_{p} ) = 0;\quad Slope_{c} (\gamma_{c} ) = 0; \hfill \\ \min (c) < c < \max (c) \hfill \\ c = p - a \hfill \\ \end{gathered} \right. \hfill \\ \end{gathered}$$

*y*^*apc*^ refers to the dependent variable that pertains to individual *i* of age *a* in period *p*, and thus belonging to the birth cohort c = p − a.

*β*_0_ denotes the constant, *X*_*j*_ is a set of covariates introduced in the model, *β*_*j*_ are the coefficients of control variables and ε_*i*_ refers to the error term. Further, *rescale(a)* and *rescale(c)* are linear functions that transform the initial values of *a* and *c* into a range between − 1 and + 1.

Finally, *α*_*a,*_* π*_*p,*_ and *γ*_*c*_ are, respectively, the detrended age, period and cohort effects. The *π*_*p*_ vector fits the categorical period changes and absorb the period-specific changes in measurements of the dependent variable. The *α*_*a*_ effects represent the non-linear age changes. For our purpose, *γ*_*c*_ effects (also named “detrended cohort effects” DCE) are the most important estimates of this model since they will detect cohort effects.

In the APCD model, if no detrended cohort effect coefficient *γ*_*c*_ is significantly different from zero, all cohorts behave according to their age and period characteristics, with no cohort-specific fluctuation. In this case, the simple age and period AP model is sufficient representation of data. However, if at least one *γ*_*c*_ coefficient is significantly different from zero, some cohorts are above or below the linear trend. Consequently, the AP model is insufficient, as some cohorts receive more or less than what one would expect, given long-run income trends.

Therefore, the APCD model shows whether a certain cohort has a more fortunate position in terms of income than other birth cohorts. However, even if later-born cohorts are below the long-run trend of income increases, they might still have a higher living standard in absolute terms (compared with former cohorts), depending on the evolution of the overall rate of income growth (Chauvel & Schröder, 2015). For instance, if overall incomes grow by 4 percent points and a cohort, for example, the 1980 cohort, has an income that grew by only 2 percent points from the previous cohort (the 1979 cohort), then the 1980 cohort is below the trend, but it is still better-off than the preceding one.

These absolute declines and progressions of income cannot be measured with the APCD model because it cannot identify linear trends due to they are equal to zero. Thus, secondly, we use the APCT-lag method (Bar-Haim et al., 2019; Chauvel et al., 2017), which shows how *absolute* earnings have developed over time. This model uses the “linear age effect” with the aim of identifying cohort trends. This implies a robust identification of the cohorts’ dynamics. In this way, this model constrains the age linear trend α to equate to the average within-cohort age effect across the cohorts in the observation window. Consider this average shift α:

3 $$\alpha = \frac{{\sum {(y_{a + 1,p + 1,c} - y^{apc} )} }}{(p - 1)(a - 1)}$$

Once α is known and the linear trend of period is constrained to zero, the cohort coefficients *γ*_*c*_ will absorb the long-term time transformations, that is, the general linear trend of social change, and make relative changes in income visible. Thus, the full model is denoted as:

4 $$\left\{ \begin{gathered} y^{apc} = \alpha_{a} + \pi_{p} + \gamma_{c} + \beta_{0} + \sum {_{j} \beta_{j} X_{j} + \varepsilon_{i} } \hfill \\ \left\{ \begin{gathered} \sum {(\alpha_{a} )} = 0;\quad \sum {(\pi_{p} )} = 0 \hfill \\ Slope(\pi_{p} ) = 0 \hfill \\ Slope(\alpha_{a} ) = \alpha = \frac{{\sum {(y_{a + 1,p + 1,c} - y^{apc} )} }}{(p - 1)(a - 1)} \hfill \\ \end{gathered} \right. \hfill \\ \min (c) < c < \max (c) \hfill \\ c = p - a \hfill \\ \end{gathered} \right.$$

## Data

### Database and Sample

Our analysis is based on the Continuous Sample of Working Histories (CSWH henceforth), conducted by the Spanish Ministry of Labour, Migration and Social Security. This data source is a random sample of around 4% of all persons who have been enrolled in affiliation or received a Social Security contributory pension at some point during the reference year. It includes rich administrative data about working lives and personal characteristics of each worker. This dataset is updated every year and allows to follow the labour market trajectories of individuals since their first affiliation to Social Security over time.

The data structure for the estimation of the models must take the form of a Lexis table, i.e. an age by period table of data with a constant pace in age and in period (e.g. 5-year age groups measured each 5 years).^4^ Thus, our key variables are age, period and birth cohort. As our dataset allows us it, we measure each age, period and cohort by 1-year interval.

Our sample covers the 2005–2019 waves of the CSWH. We select as our population of reference those individuals who have been affiliated to Social Security during the whole month of October for the relevant year i.e. from 2005 to 2019 (in line with the Wage Structure Survey, provided by the Spanish National Institute of Statistics). We focus on the private sector and on individuals who work full-time.^5^ We exclude workers younger than age 25 years and older than age 55. In both cases, the number of observations is low. Because many respondents younger than age 25 may still be in school or finishing their tertiary education, their earnings may not accurately reflect their future income potential. Therefore, we do not include these individuals in order to reduce confounding effects due to incomplete educational attainment. Similarly, we do not consider those with more than 40 years of experience giving the limited number of observations.

Hence, we focus on the cohorts of males and females born between 1950 and 1994 and analyze the inter-cohort inequality in terms of income in the period 2005–2019. Our final sample consists of 4,411,255 observations.

Our dependent variable is the logarithm of monthly earnings.^6^ The CSWH provides information on the contribution bases that are used as a proxy for income. We select the contribution base relative to the month of October. This variable is deflated using the 2016 CPI.

A set of covariates is included in the controlled model. These variables are classified into two blocks: variables related to the individual and variables related to the job. Regarding individual characteristics, we include gender, educational attainment, country of birth, nationality, region of residence and potential labour market experience. The gender is a dummy variable that takes value 1 if male and 0 otherwise. For educational attainment, we include dummy variables for having post-compulsory education, vocational training and university education. The reference group is compulsory education. The country of birth and nationality are also dummy variables that take value 1 if worker born in Spain and has the Spanish nationality, respectively, and 0 otherwise. In the case of the region of worker’ residence, we include 17 dummy variables, one for each region. Andalucia is our reference region of residence category. Potential experience is computed as the difference between the last year in which workers are observed and the year in which they entered the labour market. Hence, our period of interest covers up to 40 years of (potential) working experience.

On the other hand, variables related to the job are having a temporary contract, economic activity, contribution group and firm size. We create a dummy variable that takes value 1 if the worker has a temporary contract, 0 if he has a permanent contract. Furthermore, we include 11 dummy variables, one for each economic activity. Industry is our reference economic activity category. We do not have information relative to occupation. As a proxy, we use the contribution group, which is a 6-code variable. We include dummy variables for non-manual and medium-skilled work, non-manual and low-skilled work, manual and high-skilled work, manual and medium-skilled work, and manual and low-skilled work. The reference group is non-manual and high-skilled work. Finally, firm size is divided in six different intervals: less than 10 workers, between 10 and 19 workers, between 20 and 49 workers, between 50 and 249 workers, between 250 and 499 workers, and more than 500 workers. We create a dummy variable for each of these intervals. Our reference firm size category is less than 10 workers.

### Descriptive Statistics

**Fig. 1:**
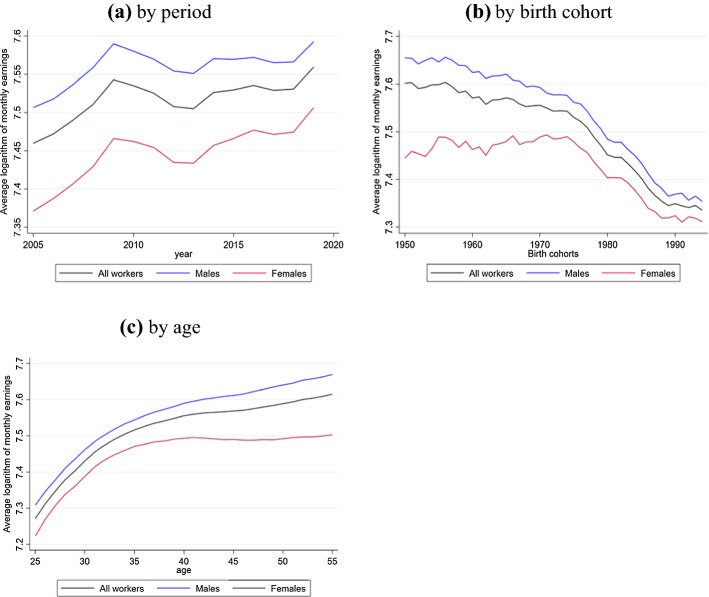
Age–Period–Cohort profile. **a** By period, **b** by birth cohort, **c** by age.* Notes*: This figure plots the average monthly earnings by age, period and birth cohort

Figure 1 illustrates the average logarithm of monthly earnings by age-period-cohort profile, distinguishing by gender. The cohort profiles show a clear downward trend in earnings level, especially from the 1973 cohort. Furthermore, the cohort profiles show different trends by gender. For women, we find an aggregate trend in which females born from 1955 to 1970 share almost the same level of earnings. For men, instead, the earnings of those born in the same period tend to decline. In both cases, the income of the cohorts born after 1973 collapses, as expected, since their transition to the job market is not yet complete.

## Results

### Age–Period–Cohort “Detrended” Model: Relative Earnings

We begin the analysis with the results of the APCD model that show whether there are differences between generations. As seen in Sect. 3, the APCD model displays relative changes in the dependent variable in relation to the linear trend-which is equal to zero-revealing which age category, period, or cohort has the most advantages compared to other groups. In our case, this allows us to answer which birth cohort has the most privileged position in terms of earnings, how age changes reveal the general effects of the life course on earnings, and whether certain shocks negatively affected earnings.

**Fig. 2:**
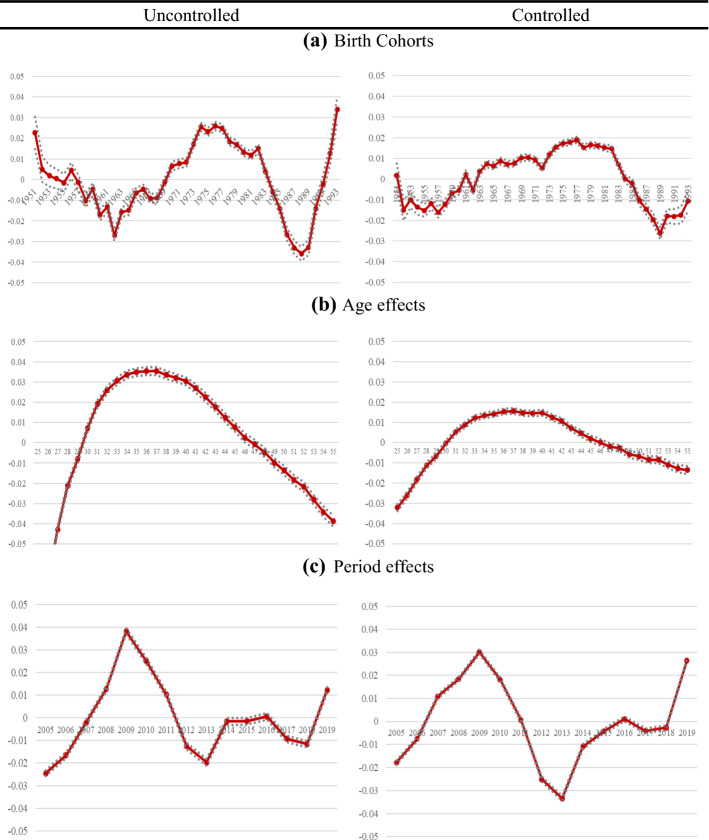
‘Detrended’ Age–Period–Cohort effects on earnings without and with controls.* Note*: Results of APCD Model (solid lines). Controlled model is adjusted by education, nationality, country of birth, region of residence, economic activity, temporary contract, experience, contribution group and firm size. Vertical axis shows APCD coefficient and horizontal axis shows each APC component. Dotted lines represent 95 percent confidence intervals

To illustrate the results of the regression, Fig. 2 shows the coefficients of the detrended effects of age–period–cohort on the earnings^7^ of all workers with and without the control variables (Table 2 in the Annex shows the coefficient estimates and their significance). Overall, we observe differences in average income between birth cohorts, age groups, and years.

First, we focus on the cohort coefficients γ_c_, since, as mentioned before, they are the most relevant coefficients in this analysis. Cohort effects show the relative effect of belonging to a given birth cohort on earnings. For instance, in the uncontrolled model the coefficient for the 1951 cohort is equal to 0.0226. Therefore, belonging to the 1951 birth cohort implies that their average earnings are 2.26 percentage points above the long-term earnings trend. The explanation is the reverse in the case that the coefficient obtained is negative. This is the case of the 1958 cohort, in which their average earnings are 1.02 percentage points below the long-term earnings trend simply because they were born that year.

Our results show statistically significant differences between generations. More specifically, before including the covariates, we note that cohorts born in the 1960s and late 1980s suffer the most in terms of income. Their average earnings are below the long-term income trend. Evidence also shows that individuals born in the late 1980s have been more negatively affected in terms of earnings than those born in the 1960s. In particular, being born in the late 1980s returns an average income approximately more than 3 percentage points below the long-term earnings trend. However, the results suggest that cohorts born in the 1970s and early 1980s, even those born in the early 1990s, are more favoured by their year of birth in terms of earnings. In other words, their average earnings are above the long-term earnings trend. Thus, belonging to the cohorts of the mid-1970s represents an average gain of more than 2 percentage points above the long-term earnings trend.

Once we control the model for covariates, the pattern is similar, although there are some differences. First, we find a smaller range of fluctuations above and below the long-term earnings trend. Second, we observe changes in which cohorts are affected positively and negatively in terms of earnings for their year of birth. We now find that cohorts born in the 1950s and early 1960s are adversely affected, that is, their average earnings are below the long-term income trend. However, younger individuals, that is, those born in the late 1980s and now also those born in the 1990s, continue to be more negatively affected. Specifically, belonging to one of these recent cohorts implies that average earnings are approximately 2 percentage points below the long-term trend.

Additionally, we observe that the number of cohorts with a privileged position in terms of earnings increases. The results show that the average incomes of the cohorts born in the 1960s, 1970s and early 1980s are above the income trend. Comparing these cohorts, we see that individuals born in the 1970s have a greater advantage than those born in the 1960s. While the average earnings of the former are approximately 2 percentage points above the long-term trend, those of the latter are about 1 percentage point higher.

Therefore, our results suggest that the baby-boomer generation (born between 1951 and 1965) as well as the Millennial generation (born between 1982 and 1993) are more affected by their year of birth in terms of earnings than generation X (1966–1981). An explanation for such results may be the economic conditions that the cohorts face in their transition to the labour market. Specifically, the first two generations begin their professional career in a context where the Spanish economy is experiencing a major economic crisis, which leads them to be in a more disadvantaged position in terms of earnings. While the baby-boomer generation faces the crisis of the 1970s and early 1980s, a crisis that affected the world and arrived late in Spain, the Millennial generation faces the financial and economic crisis of 2008 at their earliest point of transition to the labour market, in which the high rates of unemployment, especially in precarious and unstable jobs in the construction and service industries, affected them intensely. Hence, younger generations experience a deeper relative decline in income compared to those born during the baby boom. This is an important factor in income development, as we will examine in the next section.

However, the beginning of the labour market career of the generation X ranges from the late 1980s to the beginning of the 2000s. Thus, this generation begins its professional career in a context of recovery and, later, strong economic expansion, despite the early 1990s, when the economy slows down. As our results show, the generation X enjoys the most fortunate position. This generation is also experiencing an important educational expansion that, together with favourable economic conditions, offers greater job opportunities. All of this may have contributed to the positive evolution of income. In the next section, we will dig deeper into earnings growth.

Consequently, our results are related to the effects of an economic shock, supporting the idea that cohorts entering the labour market during an economic recession are in a more disadvantaged position than cohorts entering the labour market during an economic expansion (Karonen & Niemelä, 2020).

Although similar results have been found in the literature for Millennials, such as Berloffa and Villa (2010), Chauvel and Schröder (2015), Karonen and Niemelä (2020), this does not occur in the case of baby-boomers. These authors find that the baby boomer generation is in a more privileged position in terms of earnings compared to other cohorts. The reason why the baby-boom generation in the Spanish case is more negatively affected by their year of birth could be that the crisis they experienced in their transition to the labour market begins later in Spain than in the rest of Europe. In this way, the crisis is more severe during this period in Spain compared to other European labour markets. During the late 1970s, while the Spanish economy suffers a considerable increase in the unemployment rate, the reduction in employment hardly occurs in other European labour markets.

Regarding age effects, they show an inverted U-shaped convex life course, where income in mid-life ages is above the long-term income trend. The younger and older age groups, in contrast, are the most unfortunate in terms of income levels. The age coefficients show an increase in earnings once the younger cohorts enter the labour market. Furthermore, the results show a stagnation phase between the ages of 35 and 40, followed by a slowdown in earnings growth relative to the linear trend. In the controlled model, we observe that the age effects become smaller, although they keep the same shape.

Finally, the results of the period effects show the relative effect of economic fluctuations on earnings. Overall, the relative estimates underscore a clear effect linked to the Great Recession on earnings. Initially, we observe that the average earnings are above the long-term income trend due to the phase of economic expansion. This changes as a result of the origin of the economic crisis at the end of 2008. Thus, earnings are below the trend for several years, with 2013 being a turning point, although earnings keep below the long-term trend, except in 2019. The results obtained in the controlled model remain the same in general. The only thing to highlight is the most harmful effect of 2013.

In line with Karonen and Niemelä (2020), it is possible compare these period coefficients with the historical development of income inequality in the analysed country. Based on this, our findings on the period effects are in line with the evidence obtained by some authors on the evolution of income inequality in Spain. The evolution of inequality in Spain has been characterized by being strongly countercyclical (Anghel et al., 2018; Bonhomme & Hospido, 2017). Income inequality decreased substantially during the economic expansion, which, in turn, is shown as a rapid increase in the period coefficients in the APCD model. When the economic crisis began, however, income inequality increased. Hence, the decrease in the coefficients relative to the period.

In sum, the results obtained by analysing relative income (APCD model) indicate that there are differences between generations. The evidence shows that belonging to a certain age group in the labour market in certain periods leads to inequalities compared to other age groups. However, linear trends are not taken into account in this analysis, as we explained in Sect. 3. By not including them, the effect of economic growth is not attributed to successive cohorts.

Therefore, the linear trend of income growth is included in the following section to understand how income evolves from some cohorts to successive cohorts in absolute terms. Thus, we will find out if the development of earnings has stopped or slowed down for some cohorts and, in this way, show whether the economic crisis particularly affects the dynamics of absolute income of some cohorts, but not that of other cohorts.

### Age-Period-Cohort “Trended” Model: Absolute Earnings

**Fig. 3:**
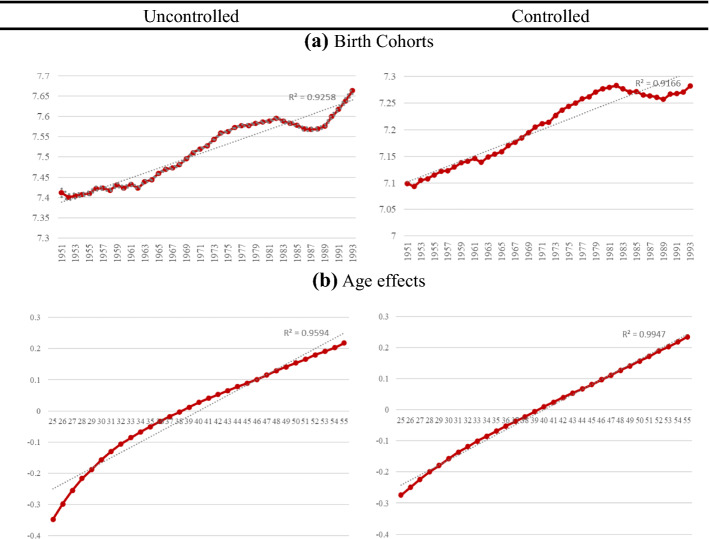
Results of APCT-Lag model without and with controls.* Note*: Results of APCT-Lag Model (solid lines). Controlled model is adjusted by education, nationality, country of birth, region of residence, economic activity, temporary contract, experience, contribution group and firm size. Vertical axis shows APCT-Lag coefficient and horizontal axis shows each APC component. As the period coefficients of the APCT-Lag model are the same as those obtained in the APCD model, we do not show them in the figure

In this second part of the analysis, we discuss the results obtained when estimating the APCT-Lag model, described in Sect. 3. This model includes the linear trends and will reveal how income develops in absolute terms across birth cohort, age, and period. In order to illustrate the results obtained, Fig. 3 shows the age–period–cohort effects for all workers before and after including the control variables (Table 3 in the Annex shows the estimates of the coefficients and their significance).

Regarding the cohort effects γ_c_, the results show statistically significant differences by cohort in the evolution of income compared to the general increase in income (the trend). In other words, the evidence suggests that there are some differences between cohort earnings growth and the trend of overall earnings growth.

First, we focus on the uncontrolled model. In Fig. 3, we observe a stagnation in the income of those workers born between the 1950s and early 1960s. However, we must highlight a substantial increase in the earnings of workers whose year of birth is between the 1960s and early 1980s, compared to the trend of general income growth. From cohorts born in the 1960s to those born in the early 1980s, earnings increase by approximately 18.8%. Much of the growth is seen among the birth cohorts of the 1960s and 1970s. For those born in the mid-70s, earnings continue to grow, but at a lower rate. Specifically, they increase at a rate similar to the general trend in income. However, for cohorts born in the 1980s, earnings decrease. The results also suggest that income growth picks up again for those born in the early 1990s.

Once we control the model with covariates, we observe in Fig. 3 that earnings growth for cohorts born in the 1950s correlates almost perfectly with overall earnings increases. Earnings grow at a similar rate to the trend, although slightly below it. Specifically, income grows at an average rate of 0.6% from a cohort born during these years to the next cohort. Looking at the growth slope of the cohorts born from the early 1960s to the early 1980s, it becomes clear that, for these cohorts, earnings increase at a roughly constant rate above the growth of overall earnings. More specifically, income grows at an average rate of 0.8% from one cohort born during these years to the next cohort. On the other hand, for the cohorts born after the 1980s, the evolution of income falls, as can be seen in the figure. Income of cohorts born in the 1980s declines by approximately 2% with an average drop of 0.4% from one cohort to another. Likewise, the earnings of cohorts born in the 1990s appear to be growing, although they are below overall earnings growth.

In sum, based on these results (APCT-Lag model) and those obtained in the previous analysis (APCD model), we observe that cohorts belonging to the baby-boom generation, despite being in a less favourable position in terms of income relative to other cohorts, are achieving a higher living standard in absolute terms than their predecessor cohorts within the baby-boom generation. As we saw before, the evolution of their income is increasing and statistically significant. In the case of cohorts born between the 1970s and early 1980s (generation X), in addition to having a more fortunate position in terms of relative earnings compared to other cohorts, they experience greater growth in their absolute earnings compared to overall income growth. Thus, cohorts born in the late 1970s have a higher living standard than those born in the early 1970s.

Conversely, our results point to a statistically significant decrease in the absolute earnings of the younger generation, that is, Millennials. Thus, the absolute living standard of these cohorts decreases. Therefore, the younger generation not only has a less favourable position in terms of relative income compared to other cohorts, but they also have a lower income in absolute terms than the predecessor cohorts. Consequently, the evidence shows that the economic crisis particularly affected the absolute income dynamics of the younger generation, but not that of other generations.

From a generational perspective, there is descriptive evidence that points to the economic crisis that began in 2008 in Spain as a turning point in the progress of workers' earnings conditions, which have been worsening over time in successive cohorts (Hernández de Cos, 2019; Puente & Regil, 2020). Thus, it is shown that, before the Great Recession, each new generation earned, on average, higher annual labour income than that of the previous one and experienced a continuous increase in hourly wages through the accumulation of work experience. Due to the economic recession, the progression in the improvement of the income of the young cohorts in comparison with the previous ones was slowed down. This led to the youngest workers being concentrated to a greater extent in the lower deciles of the wage distribution, those that correspond to the lowest incomes, and their proportion decreased in the higher wage levels.

Additionally, the evidence provided by Hernández de Cos (2019) and Puente and Regil (2020) indicates that the main causes of the slowness in the earnings recovery of young people are, on the one hand, the incidence of unemployment, especially at the beginning of the working career, and, on the other one hand, long-term unemployment with the associated loss of human capital, which would have generated a greater mismatch in the capacities of young unemployed people in the face of the labour market, especially those with a lower educational level. But also, the high rate of temporary employment presented by the current younger cohorts, which entail a loss or more difficult accumulation of experience and qualifications.

All of this illustrates that the impact of the financial and economic crisis of 2008–2013 is more adverse on the earnings of young people who are taking longer to recover. This context helps to explain the relatively high proportion of workers under 30 at risk of poverty.

Thus, our results are in line with the idea that younger generations are less favoured than members of other generations in terms of lower income and less wealth (Gale et al., 2020; Kurz et al., 2018; Rahman & Tomlinson, 2018). As Puente and Regil (2020) point out, the annual labour income of younger generations is reduced compared to that of previous generations, but this decrease seems to have a cyclical component.

Nevertheless, some authors such as Karonen and Niemelä (2020) and Chauvel and Schröder (2015) find that the level of absolute income has increased steadily from generation to generation, and no income inequalities between cohorts in absolute terms have been found. In the analysis by Chauvel and Schröder (2015), it is necessary to specify that the authors find an increase in the absolute income of the oldest to the youngest birth cohorts when they examine the American and German case, but not in the French case. In the latter, the evidence is similar to that found in our analysis. There is a stagnation in the absolute income progress of the younger cohorts.

Regarding age effects, the most notable differences are found at the extremes of the slope. In particular, it shows significant earnings growth in the youngest phase of the cohorts, when they transition to the labour market, that is, when the workers are between 25 and 30 years old. In the following years of the life cycle, earnings continue to grow in line with the trend. However, the results also show that earnings grow, but at a slower rate after 48 years.

Once we introduce the control variables, the effects of age practically overlap the trend, showing a constant increase in income throughout the life cycle. The deviations that we observe in the uncontrolled model are explained by the addition of control variables when age effects form an almost perfect line with the linear trend. These results, together with those obtained in the previous analysis, suggest that the effect of the variables included in the regression such as education, economic activity, or company size reduce the differences in all age groups. Thus, the evolution of income between age groups shows a linear trajectory in an absolute sense.

Finally, the estimated coefficients of the APCT-Lag model display the same period effects as in the APCD model. Thus, it does not provide new information on the effects of the period, so the results are not shown in the figure (to see the corresponding coefficients, see Table 3 in the Appendix).

In sum, this second approach reveals that the Millennial generation is the most disadvantaged in terms of absolute earnings. Thus, the economic crisis affects these younger cohorts more intensely compared to other cohorts, since, as we have seen, their living standard decreases.

### Gender Perspective

Using a gender perspective, we also examine possible differences between male cohorts and female cohorts in terms of relative and absolute income.

**Fig. 4:**
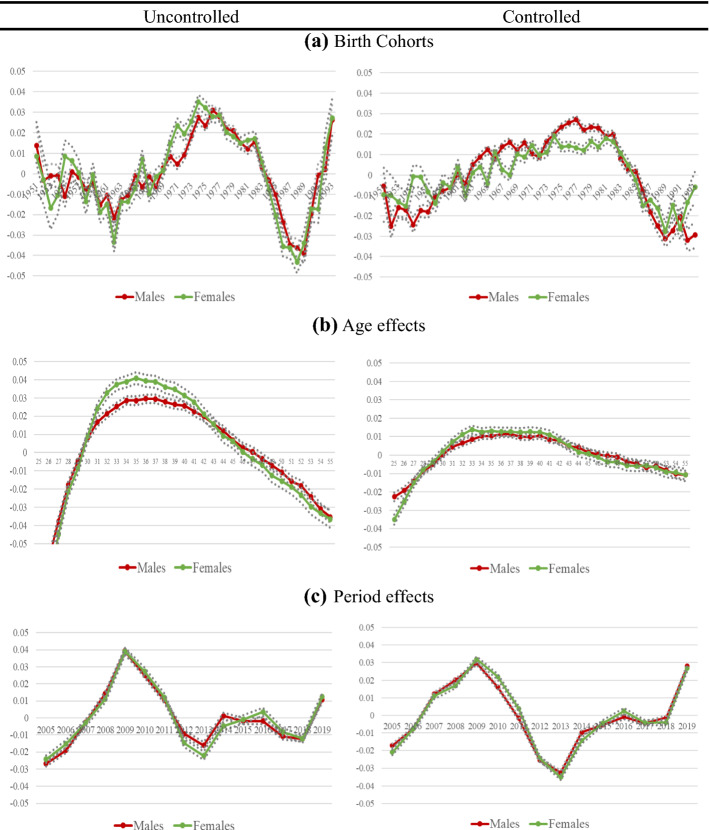
‘Detrended’ Age–Period–Cohort effects on earnings by gender without and with controls.* Note*: Results of APCD Model (solid lines). Controlled model is adjusted by education, nationality, country of birth, region of residence, economic activity, temporary contract, experience, contribution group and firm size. Vertical axis shows APCD coefficient and horizontal axis shows each APC component. Dotted lines represent 95 percent confidence intervals

In relation to relative income, the results of cohort effects on the income of men and women before and after including the control variables are consistent with what is obtained for the overall sample (see Fig. 4 and Table 2 in Annex). The generation born in the 1970s and early 1980s experiences a period of high economic growth in their transition to the labour market, as well as an expansion of educational opportunities, especially for women.^8^ Based on data published by the World Bank, while the percentage of men with secondary school enrolment rose from 50% in 1971 to 73% in 1981 and to 86% in 1999, in the case of women, this percentage rose from 42% in 1971 to 74% in 1981 and to 89% in 1999.

In the case of women of working age, the economic situation they are experiencing leads them to join the employed group with greater intensity or to look for work, although their unemployment rate remains high. Thus, the percentage of active women with respect to the total labour force rose from 27.8% in 1980 to 43% in 2008 and to 46% in 2019, based on data available from the World Bank. All this contributes to the positive evolution of income. Hence, women born in the late 1970s and early 1980s are more benefited in terms of earnings than those born in the late 1960s. Similar results are found for men.

For both genders, the results also show that the most disadvantaged cohorts are the younger generations. Despite being below the long-term income trend, female cohorts are closer to the trend than males.

**Fig. 5:**
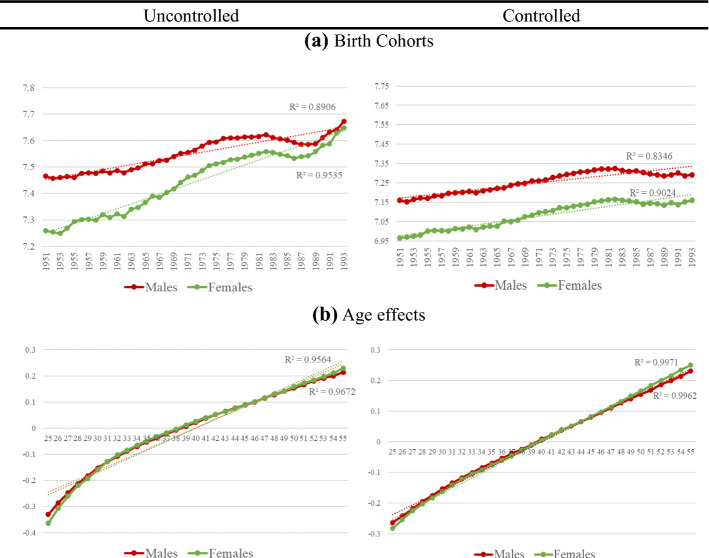
Results of APCT-Lag model without and with controls by gender.* Note*: Results of APCT-Lag Model (solid lines). Controlled model is adjusted by education, nationality, country of birth, region of residence, economic activity, temporary contract, experience, contribution group and firm size. Vertical axis shows APCT-Lag coefficient and horizontal axis shows each APC component. As the period coefficients of the APCT-Lag model are the same as those obtained in the APCD model, we do not show them in the figure

In relation to absolute earnings, for both genders there are differences between the earnings growth of the cohorts and the trend of the overall earnings growth of each gender, especially once we control the model for covariates (see Fig. 5 and Table 3 in the Appendix). This implies that for some cohorts, income growth is higher or lower than overall income growth. Focusing on the controlled model, we observe that the earnings growth of the male and female cohorts in the 1950s and early 1960s is similar to the overall increase in earnings.

On the other hand, we find that the earnings of workers born between the 1960s and the late 1970s increase at a constant rate above the growth of overall earnings, especially for women. Thus, the earnings of women increased approximately 16.6% during that period, compared to those of men, which increased 13.2%. For the cohorts born after the 1980s, on the other hand, the evolution of income stagnates in the case of women and becomes practically flat. In the case of men, earnings decrease slightly. If we compare the income growth of men with that of women, we find that the gap in terms of earnings tends to decrease slightly by birth cohort.

In sum, the evidence indicates that there are differences within female cohorts, on the one hand, and within male cohorts, on the other. The results confirm the idea that the younger cohorts, whether men or women, have a lower absolute standard of living than the predecessor cohorts. This result suggests that the economic crisis particularly affects the absolute income dynamics of the younger generation, but not that of other generations, regardless of gender.

## Conclusions

In this article, we present evidence on the possible existence of income inequality between different generations during the period 2005–2019 from two approaches: relative and absolute income. We also take into account possible gender differences. From a life course perspective, we can separately identify age, time and cohort effects. This opens up a new perspective on the income inequality topic.

From the relative perspective, our results indicate that there are differences between generations. In particular, we find that the baby-boom generation (born between 1951 and 1965) and the Millennial generation (born between 1982 and 1993) are more disadvantaged by their year of birth in terms of earnings than the generation X (1966–1981). These results in relative terms are consistent with the idea that the economic conditions faced by the cohorts in their transition to the labour market are a key factor in determining whether they are in a more or less favoured position. Thus, the generation X enters the labour market in a context of recovery and strong economic expansion, along with a development of educational opportunities. Hence, they have a more fortunate position. The baby-boom and Millennial generation, instead, start their professional career under adverse economic conditions, which leads them to be in a more disadvantaged position in terms of earnings, especially for the latter. Distinguishing by gender, the evidence also indicates that there are inequalities between female cohorts, on the one hand, and male cohorts, on the other.

From the absolute perspective, we observe again differences between generations. Particularly, the evidence indicates that the Millennial generation is the most disadvantaged in terms of absolute income. Therefore, the younger generation not only has a less favourable position in terms of relative income compared to other cohorts, but they also have a lower absolute standard of living than the predecessor cohorts. Thus, our findings show that the economic crisis particularly affected the absolute income dynamics of the referent generation, but not that of other generations. By gender, our results confirm the idea that the younger cohorts, whether males or females, have a lower absolute standard of living than the predecessor cohorts. Furthermore, if we compare the evolution of the absolute income of males versus that of females, we find that the standard of living of males is higher than that of females, despite the fact that this gap has been narrowing from generation to generation.

Our evidence presents a clear but troubling picture of the economic position of the Millennial generation in their transition to the labour market. The financial and economic crisis that began in 2008 put a significant brake on the expectations of prosperity especially for young people, while at the same time entailing significant costs and risks of a social and collective nature. Therefore, it should receive more attention from policy makers. Concretely, it seems clear the need to implement economic, educational and labour policies capable of facilitating that young people in Spain have access to jobs that allow them to acquire and accumulate professional experience, reinforcing their employability and strengthening their work paths.

This is not only important because the economic crisis has particularly affected the younger generation, placing them in a more unfavourable position in terms of earnings and reducing their living standard compared to other generations, but this may have long-term consequences which may widen existing economic disparities and limit the development of the human capital of these cohorts, which in turn has negative implications for future economic growth. In this sense, Escalonilla et al. (2021) find a significant penalty in terms of wages for young workers entering the labour market during the economic crisis, which persists several years since their entry. Therefore, it is necessary to reinforce all policies aimed at young cohorts, avoiding that, due to the accumulated effect of the crisis, a reality of low earnings can become chronic, which affects them more differentially, with negative individual and social consequences.

This situation may be aggravated in the context of the new crisis caused by COVID-19. The significance of the consequences that the already verified drop in employment and the increase in unemployment among young people may have, within a panorama of strong general deterioration in the labour market, will depend on the depth and duration of this new crisis. The prospects created by the impact of the COVID-19 pandemic are again very worrying for the younger group. The intense and rapid deterioration of their position in the labour market, and its effects in the medium and long term, increase the risk of aggravating a situation of social precariousness. The current generations of the young population, and particularly the older ones, have lived between two devastating crises practically successive that weighed down their expectations and their real options to achieve a full transition to independent adult life.

## Appendix

See Tables 1, 2 and 3.

**Table 1:** Average logarithm of monthly earnings by age and period categories

Age/period	2005	2006	2007	2008	2009	2010	2011	2012	2013	2014	2015	2016	2017	2018	2019
25	7.23	7.25	7.29	7.31	7.32	7.30	7.27	7.22	7.19	7.19	7.20	7.23	7.26	7.29	7.33
26	7.27	7.29	7.32	7.35	7.36	7.34	7.32	7.27	7.23	7.25	7.24	7.28	7.30	7.32	7.39
27	7.30	7.33	7.35	7.39	7.38	7.38	7.36	7.31	7.27	7.29	7.27	7.31	7.33	7.35	7.41
28	7.34	7.36	7.39	7.41	7.43	7.41	7.39	735	7.32	7.32	7.32	7.34	7.35	7.38	7.43
29	7.37	7.38	7.41	7.43	7.45	7.44	7.42	7.38	7.35	7.36	7.35	7.36	7.37	7.39	7.45
30	7.39	7.41	7.44	7.45	7.48	7.45	7.44	7.41	7.39	7.40	7.40	7.40	7.40	7.42	7.45
31	7.42	7.43	7.45	7.47	7.50	7.48	7.46	7.43	7.43	7.44	7.43	7.44	7.43	7.44	7.47
32	7.42	7.45	7.47	7.49	7.51	7.50	7.48	7.45	7.44	7.46	7.46	7.46	7.46	7.46	7.49
33	7.43	7.45	7.48	7.50	7.53	7.52	7.49	7.48	7.46	7.48	7.49	7.48	7.47	7.47	7.50
34	7.45	7.46	7.49	7.51	7.54	7.53	7.51	7.49	7.48	7.49	7.50	7.50	7.49	7.49	7.51
35	7.45	7.47	7.48	7.51	7.55	7.54	7.53	7.51	7.49	7.52	7.51	7.51	7.51	7.50	7.53
36	7.46	7.47	7.49	7.51	7.55	7.55	7.53	7.52	7.52	7.52	7.53	7.53	7.51	7.52	7.54
37	7.46	7.48	7.50	7.51	7.56	7.55	7.54	7.52	7.52	7.52	7.54	7.54	7.53	7.52	7.55
38	7.47	7.47	7.49	7.52	7.55	7.55	7.54	7.53	7.53	7.55	7.56	7.55	7.53	7.53	7.55
39	7.47	7.48	7.50	7.52	7.56	7.55	7.54	7.53	7.54	7.56	7.56	7.57	7.55	7.54	7.57
40	7.48	7.49	7.50	7.52	7.56	7.56	7.54	7.53	7.54	7.57	7.57	7.57	7.56	7.55	7.57
41	7.48	7.49	7.51	7.51	7.55	7.55	7.55	7.54	7.53	7.56	7.57	7.58	7.57	7.57	7.58
42	7.48	7.49	7.51	7.52	7.55	7.55	7.54	7.54	7.53	7.56	7.58	7.59	7.57	7.57	7.59
43	7.47	7.49	7.50	7.52	7.56	7.55	7.54	7.53	7.54	7.57	7.57	7.58	7.58	7.57	7.60
44	7.48	7.48	7.50	7.52	7.56	7.56	7.54	7.53	7.54	7.57	7.58	7.57	7.57	7.58	7.60
45	7.49	7.50	7.49	7.52	7.56	7.56	7.56	7.54	7.54	7.56	7.58	7.58	7.57	7.57	7.61
46	7.51	7.50	7.51	7.51	7.56	7.56	7.56	7.54	7.54	7.56	7.57	7.58	7.58	7.57	7.60
47	7.51	7.51	7.51	7.53	7.55	7.56	7.55	7.55	7.56	7.57	7.57	7.58	7.57	7.57	7.59
48	7.53	7.52	7.53	7.53	7.57	7.55	7.56	7.54	7.55	7.58	7.58	7.58	7.57	7.57	7.60
49	7.53	7.53	7.53	7.55	7.57	7.57	7.54	7.54	7.55	7.58	7.59	7.58	7.57	7.57	7.60
50	7.54	7.54	7.54	7.55	7.59	7.57	7.57	7.53	7.56	7.58	7.59	7.59	7.57	7.56	7.59
51	7.53	7.54	7.55	7.56	7.59	7.59	7.57	7.56	7.54	7.58	7.58	7.59	7.58	7.57	7.59
52	7.54	7.55	7.55	7.57	7.60	7.58	7.59	7.55	7.56	7.57	7.59	7.59	7.58	7.58	7.59
53	7.54	7.54	7.55	7.57	7.61	7.60	7.58	7.57	7.56	7.58	7.58	7.59	7.57	7.57	7.60
54	7.55	7.55	7.55	7.57	7.61	7.61	7.60	7.57	7.58	7.59	7.59	7.58	7.57	7.57	7.60
55	7.55	7.56	7.56	7.57	7.61	7.61	7.60	7.58	7.58	7.62	7.60	7.60	7.57	7.58	7.60

Birth cohorts are shown along the diagonal.

**Table 2:** Detrended age-period-cohort effects

Variables	(1)	(2)	(3)	(4)	(5)	(6)
	Model 1	Model 2	Model 3	Model 4	Model 5	Model 6
	Uncontrolled			Controlled		
	All workers	Males	Females	All workers	Males	Females
coh_1951	0.0226***	0.0138***	0.00843	0.00173	− 0.00539*	− 0.00977
	(0.00399)	(0.00436)	(0.00853)	(0.00302)	(0.00318)	(0.00676)
coh_1952	0.00490	− 0.00329	− 0.00286	− 0.0150***	− 0.0252***	− 0.00999**
	(0.00316)	(0.00357)	(0.00624)	(0.00248)	(0.00277)	(0.00494)
coh_1953	0.00185	− 0.00106	− 0.0168***	− 0.0100***	− 0.0159***	− 0.0131***
	(0.00264)	(0.00295)	(0.00524)	(0.00198)	(0.00215)	(0.00404)
coh_1954	0.000457	− 0.00102	− 0.0106**	− 0.0136***	− 0.0172***	− 0.0154***
	(0.00239)	(0.00269)	(0.00468)	(0.00179)	(0.00197)	(0.00351)
coh_1955	− 0.00146	− 0.0111***	0.00844**	− 0.0152***	− 0.0244***	− 0.000744
	(0.00211)	(0.00244)	(0.00395)	(0.00158)	(0.00183)	(0.00285)
coh_1956	0.00448**	0.000940	0.00629*	− 0.0118***	− 0.0175***	− 0.000962
	(0.00191)	(0.00219)	(0.00360)	(0.00137)	(0.00155)	(0.00252)
coh_1957	− 0.00144	− 0.00166	0.000293	− 0.0162***	− 0.0181***	− 0.00833***
	(0.00176)	(0.00203)	(0.00324)	(0.00130)	(0.00147)	(0.00238)
coh_1958	− 0.0102***	− 0.00744***	− 0.0136***	− 0.0121***	− 0.0105***	− 0.0139***
	(0.00165)	(0.00194)	(0.00295)	(0.00121)	(0.00140)	(0.00213)
coh_1959	− 0.00461***	− 0.00450**	− 0.000571	− 0.00687***	− 0.00791***	− 0.00372*
	(0.00157)	(0.00182)	(0.00287)	(0.00115)	(0.00129)	(0.00212)
coh_1960	− 0.0170***	− 0.0153***	− 0.0189***	− 0.00542***	− 0.00606***	− 0.00606***
	(0.00147)	(0.00173)	(0.00266)	(0.00108)	(0.00124)	(0.00196)
coh_1961	− 0.0132***	− 0.0107***	− 0.0150***	0.00228**	0.000414	0.00390**
	(0.00140)	(0.00164)	(0.00250)	(0.00101)	(0.00119)	(0.00176)
coh_1962	− 0.0270***	− 0.0216***	− 0.0334***	− 0.00537***	− 0.00426***	− 0.0100***
	(0.00134)	(0.00159)	(0.00236)	(0.000978)	(0.00115)	(0.00168)
coh_1963	− 0.0158***	− 0.0128***	− 0.0137***	0.00366***	0.00506***	0.00120
	(0.00127)	(0.00151)	(0.00223)	(0.000913)	(0.00107)	(0.00159)
coh_1964	− 0.0148***	− 0.0106***	− 0.0138***	0.00744***	0.00882***	0.00392***
	(0.00118)	(0.00140)	(0.00207)	(0.000845)	(0.000980)	(0.00149)
coh_1965	− 0.00645***	− 0.00112	− 0.00729***	0.00647***	0.0125***	− 0.00401***
	(0.00118)	(0.00140)	(0.00208)	(0.000848)	(0.000980)	(0.00148)
coh_1966	− 0.00470***	− 0.00647***	0.00710***	0.00888***	0.00792***	0.0114***
	(0.00116)	(0.00138)	(0.00206)	(0.000831)	(0.000970)	(0.00145)
coh_1967	− 0.00916***	− 0.00148	− 0.00982***	0.00722***	0.0139***	0.00252*
	(0.00114)	(0.00137)	(0.00198)	(0.000829)	(0.000986)	(0.00138)
coh_1968	− 0.00874***	− 0.00623***	− 0.00149	0.00779***	0.0161***	3.32e− 06
	(0.00114)	(0.00136)	(0.00199)	(0.000824)	(0.000974)	(0.00140)
coh_1969	− 0.00117	0.00283**	0.00167	0.0103***	0.0123***	0.0103***
	(0.00112)	(0.00134)	(0.00195)	(0.000808)	(0.000956)	(0.00138)
coh_1970	0.00661***	0.00826***	0.0149***	0.0106***	0.0158***	0.00879***
	(0.00109)	(0.00131)	(0.00191)	(0.000795)	(0.000938)	(0.00136)
coh_1971	0.00776***	0.00466***	0.0233***	0.00939***	0.0105***	0.0145***
	(0.00107)	(0.00129)	(0.00186)	(0.000779)	(0.000926)	(0.00132)
coh_1972	0.00847***	0.00924***	0.0193***	0.00532***	0.00873***	0.00975***
	(0.00106)	(0.00129)	(0.00183)	(0.000768)	(0.000922)	(0.00128)
coh_1973	0.0172***	0.0184***	0.0253***	0.0118***	0.0166***	0.0111***
	(0.00104)	(0.00126)	(0.00178)	(0.000750)	(0.000899)	(0.00125)
coh_1974	0.0255***	0.0272***	0.0350***	0.0156***	0.0198***	0.0191***
	(0.00101)	(0.00124)	(0.00170)	(0.000726)	(0.000886)	(0.00118)
coh_1975	0.0231***	0.0232***	0.0320***	0.0174***	0.0234***	0.0137***
	(0.000990)	(0.00121)	(0.00168)	(0.000721)	(0.000875)	(0.00118)
coh_1976	0.0259***	0.0309***	0.0277***	0.0179***	0.0255***	0.0143***
	(0.000973)	(0.00121)	(0.00162)	(0.000712)	(0.000870)	(0.00114)
coh_1977	0.0248***	0.0279***	0.0285***	0.0190***	0.0270***	0.0133***
	(0.000959)	(0.00118)	(0.00160)	(0.000714)	(0.000878)	(0.00113)
coh_1978	0.0185***	0.0216***	0.0201***	0.0153***	0.0219***	0.0121***
	(0.000952)	(0.00119)	(0.00157)	(0.000705)	(0.000877)	(0.00110)
coh_1979	0.0170***	0.0208***	0.0180***	0.0166***	0.0234***	0.0165***
	(0.000972)	(0.00122)	(0.00158)	(0.000729)	(0.000919)	(0.00112)
coh_1980	0.0133***	0.0150***	0.0148***	0.0163***	0.0229***	0.0138***
	(0.000956)	(0.00119)	(0.00156)	(0.000728)	(0.000910)	(0.00114)
coh_1981	0.0119***	0.0121***	0.0164***	0.0153***	0.0190***	0.0180***
	(0.00103)	(0.00131)	(0.00163)	(0.000777)	(0.000992)	(0.00119)
coh_1982	0.0151***	0.0157***	0.0171***	0.0149***	0.0197***	0.0163***
	(0.00108)	(0.00137)	(0.00173)	(0.000812)	(0.00104)	(0.00123)
coh_1983	0.00391***	0.00312**	0.00564***	0.00721***	0.00795***	0.0107***
	(0.00118)	(0.00150)	(0.00190)	(0.000897)	(0.00115)	(0.00137)
coh_1984	− 0.00582***	− 0.00309*	− 0.00799***	− 5.94e-05	0.00264**	0.00497***
	(0.00128)	(0.00164)	(0.00202)	(0.000976)	(0.00126)	(0.00147)
coh_1985	− 0.0141***	− 0.0105***	− 0.0206***	− 0.00200*	0.00179	− 0.00241
	(0.00139)	(0.00179)	(0.00219)	(0.00106)	(0.00137)	(0.00160)
coh_1986	− 0.0266***	− 0.0236***	− 0.0356***	− 0.0103***	− 0.00788***	− 0.0151***
	(0.00153)	(0.00194)	(0.00245)	(0.00116)	(0.00149)	(0.00176)
coh_1987	− 0.0330***	− 0.0348***	− 0.0363***	− 0.0146***	− 0.0181***	− 0.0121***
	(0.00164)	(0.00209)	(0.00262)	(0.00125)	(0.00164)	(0.00188)
coh_1988	− 0.0358***	− 0.0362***	− 0.0432***	− 0.0196***	− 0.0249***	− 0.0163***
	(0.00177)	(0.00225)	(0.00283)	(0.00135)	(0.00176)	(0.00202)
coh_1989	− 0.0327***	− 0.0389***	− 0.0340***	− 0.0262***	− 0.0313***	− 0.0278***
	(0.00191)	(0.00243)	(0.00305)	(0.00146)	(0.00192)	(0.00218)
coh_1990	− 0.0140***	− 0.0197***	− 0.0172***	− 0.0179***	− 0.0272***	− 0.0147***
	(0.00206)	(0.00266)	(0.00324)	(0.00159)	(0.00211)	(0.00237)
coh_1991	− 0.00231	− 0.000703	− 0.0172***	− 0.0181***	− 0.0204***	− 0.0264***
	(0.00226)	(0.00291)	(0.00355)	(0.00179)	(0.00234)	(0.00269)
coh_1992	0.0128***	0.00210	0.0125***	− 0.0174***	− 0.0320***	− 0.0133***
	(0.00251)	(0.00324)	(0.00395)	(0.00205)	(0.00269)	(0.00310)
coh_1993	0.0339***	0.0262***	0.0270***	− 0.0107***	− 0.0292***	− 0.00589
	(0.00305)	(0.00387)	(0.00489)	(0.00253)	(0.00332)	(0.00383)
age_0025	− 0.0985***	− 0.0842***	− 0.114***	− 0.0321***	− 0.0226***	− 0.0350***
	(0.00110)	(0.00139)	(0.00175)	(0.000914)	(0.00119)	(0.00136)
age_0026	− 0.0676***	− 0.0582***	− 0.0751***	− 0.0259***	− 0.0193***	− 0.0253***
	(0.00103)	(0.00130)	(0.00164)	(0.000837)	(0.00108)	(0.00125)
age_0027	− 0.0428***	− 0.0375***	− 0.0449***	− 0.0183***	− 0.0143***	− 0.0151***
	(0.000989)	(0.00125)	(0.00157)	(0.000789)	(0.00102)	(0.00118)
age_0028	− 0.0211***	− 0.0176***	− 0.0211***	− 0.0113***	− 0.00858***	− 0.00784***
	(0.000964)	(0.00122)	(0.00153)	(0.000751)	(0.000968)	(0.00112)
age_0029	− 0.00803***	− 0.00471***	− 0.00856***	− 0.00672***	− 0.00493***	− 0.00364***
	(0.000956)	(0.00121)	(0.00152)	(0.000735)	(0.000944)	(0.00111)
age_0030	0.00724***	0.00807***	0.00892***	− 0.000437	0.000290	0.00197*
	(0.000945)	(0.00120)	(0.00151)	(0.000721)	(0.000917)	(0.00110)
age_0031	0.0191***	0.0167***	0.0240***	0.00513***	0.00452***	0.00713***
	(0.000941)	(0.00119)	(0.00152)	(0.000710)	(0.000896)	(0.00109)
age_0032	0.0261***	0.0214***	0.0328***	0.00900***	0.00646***	0.0111***
	(0.000941)	(0.00118)	(0.00154)	(0.000701)	(0.000876)	(0.00109)
age_0033	0.0307***	0.0252***	0.0373***	0.0121***	0.00851***	0.0138***
	(0.000947)	(0.00118)	(0.00156)	(0.000698)	(0.000867)	(0.00110)
age_0034	0.0337***	0.0285***	0.0388***	0.0134***	0.0104***	0.0125***
	(0.000957)	(0.00119)	(0.00159)	(0.000700)	(0.000863)	(0.00111)
age_0035	0.0350***	0.0285***	0.0409***	0.0143***	0.0103***	0.0130***
	(0.000970)	(0.00119)	(0.00163)	(0.000707)	(0.000863)	(0.00114)
age_0036	0.0354***	0.0295***	0.0393***	0.0154***	0.0114***	0.0127***
	(0.000979)	(0.00119)	(0.00167)	(0.000709)	(0.000858)	(0.00116)
age_0037	0.0355***	0.0295***	0.0388***	0.0157***	0.0114***	0.0126***
	(0.000989)	(0.00120)	(0.00170)	(0.000715)	(0.000858)	(0.00118)
age_0038	0.0335***	0.0278***	0.0360***	0.0147***	0.00994***	0.0124***
	(0.00100)	(0.00122)	(0.00174)	(0.000727)	(0.000871)	(0.00121)
age_0039	0.0321***	0.0263***	0.0347***	0.0145***	0.00976***	0.0127***
	(0.00101)	(0.00122)	(0.00176)	(0.000727)	(0.000869)	(0.00121)
age_0040	0.0305***	0.0257***	0.0313***	0.0148***	0.0105***	0.0124***
	(0.00102)	(0.00123)	(0.00178)	(0.000732)	(0.000871)	(0.00123)
age_0041	0.0270***	0.0225***	0.0277***	0.0125***	0.00842***	0.0110***
	(0.00104)	(0.00125)	(0.00180)	(0.000740)	(0.000878)	(0.00125)
age_0042	0.0225***	0.0197***	0.0209***	0.0106***	0.00782***	0.00810***
	(0.00105)	(0.00126)	(0.00183)	(0.000751)	(0.000886)	(0.00127)
age_0043	0.0176***	0.0156***	0.0155***	0.00709***	0.00492***	0.00509***
	(0.00106)	(0.00128)	(0.00185)	(0.000759)	(0.000898)	(0.00128)
age_0044	0.0124***	0.0120***	0.00901***	0.00454***	0.00402***	0.00161
	(0.00108)	(0.00130)	(0.00188)	(0.000775)	(0.000915)	(0.00131)
age_0045	0.00764***	0.00680***	0.00615***	0.00190**	0.00148	0.000655
	(0.00110)	(0.00132)	(0.00190)	(0.000787)	(0.000932)	(0.00133)
age_0046	0.00247**	0.00274**	0.000474	0.000107	0.000492	− 0.00110
	(0.00111)	(0.00133)	(0.00192)	(0.000794)	(0.000936)	(0.00135)
age_0047	− 0.000819	0.000489	− 0.00340*	− 0.00195**	− 0.000369	− 0.00371***
	(0.00112)	(0.00135)	(0.00195)	(0.000806)	(0.000946)	(0.00137)
age_0048	− 0.00490***	− 0.00331**	− 0.00682***	− 0.00273***	− 0.000930	− 0.00385***
	(0.00114)	(0.00136)	(0.00197)	(0.000813)	(0.000954)	(0.00139)
age_0049	− 0.00982***	− 0.00695***	− 0.0125***	− 0.00580***	− 0.00362***	− 0.00559***
	(0.00116)	(0.00139)	(0.00200)	(0.000831)	(0.000979)	(0.00141)
age_0050	− 0.0136***	− 0.0106***	− 0.0155***	− 0.00679***	− 0.00453***	− 0.00584***
	(0.00118)	(0.00141)	(0.00203)	(0.000838)	(0.000992)	(0.00141)
age_0051	− 0.0183***	− 0.0156***	− 0.0188***	− 0.00842***	− 0.00675***	− 0.00563***
	(0.00120)	(0.00144)	(0.00209)	(0.000856)	(0.00101)	(0.00144)
age_0052	− 0.0218***	− 0.0181***	− 0.0232***	− 0.00842***	− 0.00580***	− 0.00684***
	(0.00122)	(0.00145)	(0.00213)	(0.000870)	(0.00102)	(0.00149)
age_0053	− 0.0281***	− 0.0242***	− 0.0296***	− 0.0108***	− 0.00805***	− 0.00905***
	(0.00126)	(0.00149)	(0.00221)	(0.000896)	(0.00105)	(0.00155)
age_0054	− 0.0342***	− 0.0309***	− 0.0331***	− 0.0127***	− 0.0101***	− 0.00943***
	(0.00130)	(0.00155)	(0.00229)	(0.000935)	(0.00110)	(0.00162)
age_0055	− 0.0388***	− 0.0352***	− 0.0364***	− 0.0135***	− 0.0107***	− 0.0107***
	(0.00141)	(0.00167)	(0.00246)	(0.00100)	(0.00118)	(0.00172)
per_2005	− 0.0245***	− 0.0268***	− 0.0243***	− 0.0179***	− 0.0172***	− 0.0210***
	(0.000641)	(0.000763)	(0.00113)	(0.000495)	(0.000577)	(0.000857)
per_2006	− 0.0165***	− 0.0190***	− 0.0153***	− 0.00760***	− 0.00758***	− 0.00760***
	(0.000634)	(0.000758)	(0.00111)	(0.000479)	(0.000563)	(0.000817)
per_2007	− 0.00215***	− 0.00274***	− 0.00291***	0.0110***	0.0121***	0.0110***
	(0.000633)	(0.000759)	(0.00110)	(0.000510)	(0.000604)	(0.000855)
per_2008	0.0128***	0.0143***	0.0112***	0.0184***	0.0200***	0.0169***
	(0.000642)	(0.000773)	(0.00110)	(0.000476)	(0.000565)	(0.000789)
per_2009	0.0383***	0.0393***	0.0390***	0.0302***	0.0297***	0.0320***
	(0.000677)	(0.000823)	(0.00114)	(0.000493)	(0.000594)	(0.000802)
per_2010	0.0250***	0.0250***	0.0273***	0.0182***	0.0161***	0.0219***
	(0.000692)	(0.000847)	(0.00116)	(0.000503)	(0.000610)	(0.000812)
per_2011	0.0102***	0.0104***	0.0120***	0.000569	− 0.00160***	0.00394***
	(0.000707)	(0.000871)	(0.00118)	(0.000510)	(0.000622)	(0.000818)
per_2012	− 0.0124***	− 0.00929***	− 0.0146***	− 0.0250***	− 0.0255***	− 0.0244***
	(0.000735)	(0.000911)	(0.00121)	(0.000530)	(0.000654)	(0.000838)
per_2013	− 0.0196***	− 0.0161***	− 0.0223***	− 0.0334***	− 0.0326***	− 0.0350***
	(0.000781)	(0.000973)	(0.00127)	(0.000559)	(0.000694)	(0.000880)
per_2014	− 0.00160**	0.00117	− 0.00470***	− 0.0109***	− 0.00966***	− 0.0144***
	(0.000776)	(0.000964)	(0.00128)	(0.000553)	(0.000681)	(0.000883)
per_2015	− 0.00146*	− 0.00186**	− 0.000939	− 0.00432***	− 0.00539***	− 0.00445***
	(0.000757)	(0.000940)	(0.00125)	(0.000535)	(0.000660)	(0.000854)
per_2016	0.000648	− 0.00181**	0.00355***	0.00108**	− 0.000876	0.00254***
	(0.000733)	(0.000910)	(0.00121)	(0.000519)	(0.000640)	(0.000829)
per_2017	− 0.00930***	− 0.0110***	− 0.00824***	− 0.00415***	− 0.00442***	− 0.00417***
	(0.000708)	(0.000873)	(0.00118)	(0.000504)	(0.000616)	(0.000816)
per_2018	− 0.0116***	− 0.0123***	− 0.0122***	− 0.00266***	− 0.00119**	− 0.00407***
	(0.000680)	(0.000839)	(0.00114)	(0.000489)	(0.000598)	(0.000792)
per_2019	0.0122***	0.0107***	0.0125***	0.0266***	0.0280***	0.0267***
	(0.000654)	(0.000809)	(0.00109)	(0.000466)	(0.000573)	(0.000749)
rescacoh	0.0670***	0.0367***	0.159***	− 0.0628***	− 0.0690***	− 0.0617***
	(0.00142)	(0.00170)	(0.00252)	(0.00110)	(0.00131)	(0.00192)
rescaage	0.176***	0.175***	0.179***	0.00664***	0.00993***	0.0332***
	(0.000735)	(0.000892)	(0.00126)	(0.000766)	(0.000968)	(0.00120)
_Ispanish_n_1				− 0.0128***	0.00634***	− 0.0144***
				(0.000864)	(0.00109)	(0.00138)
_Iabroad_1				− 0.0233***	− 0.0279***	− 0.0252***
				(0.000733)	(0.000941)	(0.00111)
_Ivocationa_1				0.0935***	0.0926***	0.0962***
				(0.000379)	(0.000457)	(0.000624)
_Ipostcompu_1				0.0797***	0.0740***	0.0950***
				(0.000536)	(0.000682)	(0.000823)
_Iuniversit_1				0.149***	0.140***	0.175***
				(0.000540)	(0.000723)	(0.000800)
_Iccaa_resi_2				0.0353***	0.0591***	0.0176***
				(0.000886)	(0.00106)	(0.00144)
_Iccaa_resi_3				0.0184***	0.0572***	− 0.0365***
				(0.00104)	(0.00123)	(0.00165)
_Iccaa_resi_4				0.0672***	0.0683***	0.0932***
				(0.000904)	(0.00115)	(0.00140)
_Iccaa_resi_5				− 0.0633***	− 0.0731***	− 0.0317***
				(0.000771)	(0.000975)	(0.00120)
_Iccaa_resi_6				0.0256***	0.0427***	0.00526**
				(0.00133)	(0.00163)	(0.00208)
_Iccaa_resi_7				− 0.00661***	− 0.00307***	− 0.00156
				(0.000788)	(0.000923)	(0.00139)
_Iccaa_resi_8				8.42e–05	0.00999***	− 0.00380***
				(0.000705)	(0.000850)	(0.00117)
_Iccaa_resi_9				0.0857***	0.107***	0.0809***
				(0.000524)	(0.000626)	(0.000883)
_Iccaa_resi_10				− 0.0815***	− 0.0929***	− 0.0573***
				(0.00116)	(0.00137)	(0.00201)
_Iccaa_resi_11				− 0.0563***	− 0.0376***	− 0.0559***
				(0.000689)	(0.000830)	(0.00112)
_Iccaa_resi_12				0.0324***	0.0399***	0.0357***
				(0.000534)	(0.000656)	(0.000878)
_Iccaa_resi_13				− 0.0225***	− 0.0183***	− 0.0229***
				(0.000943)	(0.00109)	(0.00166)
_Iccaa_resi_14				0.142***	0.164***	0.119***
				(0.00111)	(0.00132)	(0.00183)
_Iccaa_resi_15				0.174***	0.182***	0.169***
				(0.000709)	(0.000850)	(0.00119)
_Iccaa_resi_16				0.0473***	0.0620***	0.0525***
				(0.00157)	(0.00188)	(0.00257)
_Iccaa_resi_17				− 0.00901***	− 0.00340***	− 0.000909
				(0.000594)	(0.000718)	(0.000985)
_Iccaa_resi_18				0.0930***	0.0748***	0.127***
				(0.00324)	(0.00395)	(0.00549)
_Itemporary_1				− 0.0909***	− 0.107***	− 0.0689***
				(0.000368)	(0.000460)	(0.000591)
exp				0.00542***	0.00462***	0.00358***
				(3.60e-05)	(4.77e-05)	(5.32e-05)
_Iagricultu_1				− 0.138***	− 0.154***	− 0.117***
				(0.00178)	(0.00189)	(0.00450)
_Iconstruct_1				− 0.00579***	− 0.0211***	− 0.0242***
				(0.000508)	(0.000545)	(0.00154)
_Itrade_1				− 0.159***	− 0.128***	− 0.151***
				(0.000474)	(0.000558)	(0.000830)
_Itourism_1				− 0.177***	− 0.185***	− 0.0671***
				(0.000590)	(0.000795)	(0.000955)
_Itransp_1				− 0.0596***	− 0.0737***	− 0.0281***
				(0.000652)	(0.000707)	(0.00150)
_Ihealth_1				− 0.202***	− 0.171***	− 0.141***
				(0.000626)	(0.00112)	(0.000877)
_Ieducat_1				− 0.274***	− 0.258***	− 0.213***
				(0.000976)	(0.00166)	(0.00128)
_Ifinancial_1				0.134***	0.105***	0.212***
				(0.000699)	(0.000858)	(0.00116)
_Is_company_1				− 0.157***	− 0.155***	− 0.111***
				(0.000525)	(0.000662)	(0.000877)
_Io_service_1				− 0.142***	− 0.112***	− 0.132***
				(0.000674)	(0.000823)	(0.00109)
_Imedium_ma_1				0.100***	0.0950***	0.0827***
				(0.000590)	(0.000694)	(0.000990)
_Ihigh_manu_1				0.186***	0.157***	0.141***
				(0.000523)	(0.000601)	(0.00105)
_Ilow_nonma_1				0.0764***	0.0601***	0.143***
				(0.000605)	(0.000860)	(0.000899)
_Imedium_no_1				0.331***	0.334***	0.350***
				(0.000588)	(0.000734)	(0.000929)
_Ihigh_nonm_1				0.563***	0.520***	0.615***
				(0.000686)	(0.000878)	(0.00107)
_Ib10_19_1				0.0749***	0.0653***	0.0774***
				(0.000510)	(0.000598)	(0.000894)
_Ib20_49_1				0.120***	0.115***	0.115***
				(0.000467)	(0.000553)	(0.000804)
_Ib50_249_1				0.188***	0.192***	0.173***
				(0.000439)	(0.000534)	(0.000723)
_Ib250_499_1				0.247***	0.254***	0.233***
				(0.000588)	(0.000733)	(0.000923)
_Imore500_1				0.286***	0.290***	0.274***
				(0.000496)	(0.000634)	(0.000759)
Constant	7.515***	7.552***	7.444***	7.080***	7.123***	6.978***
	(0.000280)	(0.000335)	(0.000505)	(0.00123)	(0.00152)	(0.00202)
Observations	4,391,751	2,739,343	1,652,408	4,230,136	2,627,941	1,602,195

This table displays the detrended age-period-cohort effects using as dependent variable the logarithm of monthly earnings. Robust standard errors in parentheses ****p* < 0.01, ***p* < 0.05, **p* < 0.1.

**Table 3:** Trended age-period-cohort effects

Variables	(1)	(2)	(3)	(4)	(5)	(6)
	Model 1	Model 2	Model 3	Model 4	Model 5	Model 6
	Uncontrolled			Controlled		
	All workers	Males	Females	All workers	Males	Females
coh_1951	7.408***	7.462***	7.262***	7.097***	7.159***	6.964***
	(0.00430)	(0.00472)	(0.00887)	(0.00365)	(0.00390)	(0.00809)
coh_1952	7.400***	7.449***	7.252***	7.092***	7.148***	6.972***
	(0.00343)	(0.00374)	(0.00657)	(0.00296)	(0.00353)	(0.00586)
coh_1953	7.406***	7.452***	7.252***	7.099***	7.166***	6.974***
	(0.00279)	(0.00312)	(0.00560)	(0.00251)	(0.00280)	(0.00486)
coh_1954	7.406***	7.463***	7.268***	7.106***	7.176***	6.985***
	(0.00251)	(0.00284)	(0.00489)	(0.00233)	(0.00266)	(0.00426)
coh_1955	7.412***	7.460***	7.294***	7.111***	7.169***	6.999***
	(0.00220)	(0.00253)	(0.00411)	(0.00213)	(0.00250)	(0.00365)
coh_1956	7.424***	7.470***	7.301***	7.121***	7.183***	7.007***
	(0.00196)	(0.00224)	(0.00373)	(0.00193)	(0.00227)	(0.00334)
coh_1957	7.424***	7.475***	7.306***	7.119***	7.186***	6.999***
	(0.00177)	(0.00203)	(0.00326)	(0.00186)	(0.00219)	(0.00322)
coh_1958	7.420***	7.474***	7.298***	7.127***	7.195***	7.000***
	(0.00166)	(0.00195)	(0.00289)	(0.00177)	(0.00214)	(0.00300)
coh_1959	7.435***	7.482***	7.322***	7.135***	7.201***	7.011***
	(0.00157)	(0.00182)	(0.00284)	(0.00172)	(0.00204)	(0.00300)
coh_1960	7.425***	7.479***	7.311***	7.139***	7.202***	7.012***
	(0.00145)	(0.00170)	(0.00264)	(0.00166)	(0.00200)	(0.00290)
coh_1961	7.436***	7.486***	7.322***	7.144***	7.209***	7.020***
	(0.00138)	(0.00162)	(0.00243)	(0.00161)	(0.00194)	(0.00268)
coh_1962	7.424***	7.479***	7.317***	7.137***	7.200***	7.010***
	(0.00132)	(0.00156)	(0.00230)	(0.00158)	(0.00192)	(0.00263)
coh_1963	7.440***	7.488***	7.342***	7.145***	7.211***	7.021***
	(0.00124)	(0.00148)	(0.00215)	(0.00153)	(0.00186)	(0.00255)
coh_1964	7.444***	7.496***	7.350***	7.152***	7.215***	7.025***
	(0.00114)	(0.00137)	(0.00198)	(0.00149)	(0.00179)	(0.00250)
coh_1965	7.461***	7.513***	7.363***	7.158***	7.223***	7.025***
	(0.00114)	(0.00136)	(0.00199)	(0.00147)	(0.00178)	(0.00248)
coh_1966	7.471***	7.512***	7.392***	7.169***	7.225***	7.051***
	(0.00113)	(0.00135)	(0.00196)	(0.00146)	(0.00176)	(0.00245)
coh_1967	7.473***	7.523***	7.387***	7.174***	7.238***	7.049***
	(0.00111)	(0.00134)	(0.00188)	(0.00145)	(0.00176)	(0.00240)
coh_1968	7.483***	7.525***	7.402***	7.183***	7.245***	7.057***
	(0.00111)	(0.00133)	(0.00190)	(0.00144)	(0.00174)	(0.00240)
coh_1969	7.498***	7.542***	7.419***	7.193***	7.250***	7.074***
	(0.00109)	(0.00131)	(0.00187)	(0.00142)	(0.00172)	(0.00238)
coh_1970	7.511***	7.553***	7.441***	7.203***	7.260***	7.083***
	(0.00106)	(0.00128)	(0.00185)	(0.00140)	(0.00170)	(0.00237)
coh_1971	7.521***	7.555***	7.461***	7.210***	7.263***	7.095***
	(0.00104)	(0.00126)	(0.00180)	(0.00139)	(0.00168)	(0.00233)
coh_1972	7.527***	7.566***	7.468***	7.214***	7.266***	7.099***
	(0.00103)	(0.00126)	(0.00176)	(0.00138)	(0.00168)	(0.00230)
coh_1973	7.543***	7.580***	7.483***	7.226***	7.278***	7.109***
	(0.00101)	(0.00122)	(0.00171)	(0.00136)	(0.00166)	(0.00227)
coh_1974	7.561***	7.594***	7.508***	7.236***	7.288***	7.122***
	(0.000975)	(0.00120)	(0.00164)	(0.00134)	(0.00164)	(0.00223)
coh_1975	7.563***	7.596***	7.514***	7.242***	7.294***	7.121***
	(0.000955)	(0.00117)	(0.00161)	(0.00133)	(0.00162)	(0.00222)
coh_1976	7.573***	7.613***	7.518***	7.249***	7.300***	7.129***
	(0.000944)	(0.00117)	(0.00155)	(0.00132)	(0.00161)	(0.00219)
coh_1977	7.577***	7.613***	7.528***	7.256***	7.311***	7.133***
	(0.000922)	(0.00114)	(0.00153)	(0.00131)	(0.00160)	(0.00219)
coh_1978	7.577***	7.611***	7.527***	7.260***	7.308***	7.137***
	(0.000917)	(0.00115)	(0.00151)	(0.00131)	(0.00160)	(0.00217)
coh_1979	7.581***	7.614***	7.538***	7.268***	7.317***	7.152***
	(0.000937)	(0.00119)	(0.00152)	(0.00132)	(0.00163)	(0.00217)
coh_1980	7.585***	7.615***	7.545***	7.274***	7.325***	7.155***
	(0.000920)	(0.00115)	(0.00152)	(0.00132)	(0.00161)	(0.00217)
coh_1981	7.590***	7.615***	7.553***	7.278***	7.323***	7.161***
	(0.00100)	(0.00127)	(0.00158)	(0.00134)	(0.00166)	(0.00220)
coh_1982	7.596***	7.624***	7.560***	7.280***	7.327***	7.165***
	(0.00107)	(0.00135)	(0.00171)	(0.00137)	(0.00169)	(0.00223)
coh_1983	7.586***	7.614***	7.553***	7.276***	7.318***	7.160***
	(0.00117)	(0.00149)	(0.00188)	(0.00142)	(0.00177)	(0.00231)
coh_1984	7.582***	7.609***	7.552***	7.269***	7.310***	7.157***
	(0.00129)	(0.00164)	(0.00203)	(0.00148)	(0.00184)	(0.00238)
coh_1985	7.577***	7.607***	7.545***	7.269***	7.313***	7.152***
	(0.00140)	(0.00180)	(0.00218)	(0.00154)	(0.00193)	(0.00245)
coh_1986	7.565***	7.599***	7.532***	7.264***	7.306***	7.139***
	(0.00155)	(0.00197)	(0.00248)	(0.00162)	(0.00201)	(0.00258)
coh_1987	7.565***	7.589***	7.538***	7.263***	7.297***	7.145***
	(0.00167)	(0.00212)	(0.00268)	(0.00169)	(0.00215)	(0.00268)
coh_1988	7.568***	7.591***	7.541***	7.260***	7.294***	7.143***
	(0.00181)	(0.00232)	(0.00289)	(0.00178)	(0.00227)	(0.00279)
coh_1989	7.573***	7.589***	7.553***	7.255***	7.286***	7.131***
	(0.00197)	(0.00254)	(0.00316)	(0.00188)	(0.00241)	(0.00289)
coh_1990	7.600***	7.610***	7.584***	7.266***	7.294***	7.149***
	(0.00215)	(0.00276)	(0.00337)	(0.00202)	(0.00260)	(0.00312)
coh_1991	7.617***	7.633***	7.587***	7.265***	7.304***	7.136***
	(0.00237)	(0.00313)	(0.00371)	(0.00221)	(0.00279)	(0.00345)
coh_1992	7.639***	7.649***	7.622***	7.271***	7.291***	7.148***
	(0.00269)	(0.00342)	(0.00418)	(0.00242)	(0.00319)	(0.00384)
coh_1993	7.662***	7.676***	7.645***	7.278***	7.292***	7.160***
	(0.00326)	(0.00415)	(0.00547)	(0.00300)	(0.00388)	(0.00451)
age_0025	− 0.346***	− 0.334***	− 0.362***	− 0.277***	− 0.266***	− 0.281***
	(0.00110)	(0.00140)	(0.00174)	(0.000937)	(0.00119)	(0.00137)
age_0026	− 0.296***	− 0.290***	− 0.307***	− 0.250***	− 0.242***	− 0.253***
	(0.00103)	(0.00131)	(0.00163)	(0.000846)	(0.00109)	(0.00128)
age_0027	− 0.253***	− 0.251***	− 0.258***	− 0.224***	− 0.217***	− 0.226***
	(0.000988)	(0.00126)	(0.00157)	(0.000797)	(0.00104)	(0.00118)
age_0028	− 0.217***	− 0.215***	− 0.220***	− 0.201***	− 0.197***	− 0.201***
	(0.000964)	(0.00123)	(0.00154)	(0.000760)	(0.000973)	(0.00114)
age_0029	− 0.186***	− 0.184***	− 0.192***	− 0.181***	− 0.178***	− 0.181***
	(0.000956)	(0.00121)	(0.00152)	(0.000741)	(0.000953)	(0.00112)
age_0030	− 0.156***	− 0.155***	− 0.158***	− 0.159***	− 0.152***	− 0.160***
	(0.000946)	(0.00120)	(0.00152)	(0.000728)	(0.000915)	(0.00110)
age_0031	− 0.129***	− 0.131***	− 0.128***	− 0.137***	− 0.137***	− 0.141***
	(0.000943)	(0.00119)	(0.00152)	(0.000713)	(0.000903)	(0.00109)
age_0032	− 0.106***	− 0.109***	− 0.103***	− 0.119***	− 0.118***	− 0.123***
	(0.000944)	(0.00118)	(0.00154)	(0.000709)	(0.000879)	(0.00110)
age_0033	− 0.0838***	− 0.0902***	− 0.0843***	− 0.102***	− 0.101***	− 0.105***
	(0.000947)	(0.00118)	(0.00155)	(0.000700)	(0.000877)	(0.00111)
age_0034	− 0.0674***	− 0.0717***	− 0.0658***	− 0.0854***	− 0.0838***	− 0.0910***
	(0.000957)	(0.00119)	(0.00160)	(0.000704)	(0.000865)	(0.00112)
age_0035	− 0.0510***	− 0.0548***	− 0.0499***	− 0.0692***	− 0.0685***	− 0.0750***
	(0.000970)	(0.00119)	(0.00163)	(0.000711)	(0.000864)	(0.00115)
age_0036	− 0.0334***	− 0.0392***	− 0.0317***	− 0.0533***	− 0.0536***	− 0.0636***
	(0.000978)	(0.00119)	(0.00167)	(0.000716)	(0.000865)	(0.00117)
age_0037	− 0.0185***	− 0.0229***	− 0.0146***	− 0.0380***	− 0.0398***	− 0.0451***
	(0.000995)	(0.00120)	(0.00169)	(0.000725)	(0.000866)	(0.00119)
age_0038	− 0.00527***	− 0.00902***	− 0.00545***	− 0.0232***	− 0.0251***	− 0.0255***
	(0.00101)	(0.00122)	(0.00174)	(0.000731)	(0.000881)	(0.00120)
age_0039	0.0119***	0.00570***	0.0133***	− 0.00699***	− 0.00960***	− 0.0107***
	(0.00102)	(0.00123)	(0.00175)	(0.000736)	(0.000883)	(0.00122)
age_0040	0.0259***	0.0223***	0.0258***	0.00894***	0.00806***	0.00508***
	(0.00103)	(0.00123)	(0.00178)	(0.000745)	(0.000883)	(0.00124)
age_0041	0.0397***	0.0369***	0.0427***	0.0248***	0.0223***	0.0224***
	(0.00104)	(0.00125)	(0.00179)	(0.000746)	(0.000886)	(0.00126)
age_0042	0.0535***	0.0516***	0.0517***	0.0391***	0.0376***	0.0375***
	(0.00105)	(0.00126)	(0.00183)	(0.000761)	(0.000901)	(0.00129)
age_0043	0.0644***	0.0631***	0.0580***	0.0527***	0.0545***	0.0493***
	(0.00107)	(0.00128)	(0.00185)	(0.000771)	(0.000909)	(0.00130)
age_0044	0.0783***	0.0763***	0.0720***	0.0678***	0.0665***	0.0644***
	(0.00109)	(0.00130)	(0.00189)	(0.000782)	(0.000928)	(0.00133)
age_0045	0.0890***	0.0889***	0.0888***	0.0810***	0.0792***	0.0799***
	(0.00110)	(0.00132)	(0.00190)	(0.000800)	(0.000954)	(0.00135)
age_0046	0.100***	0.0992***	0.0993***	0.0971***	0.0942***	0.0975***
	(0.00111)	(0.00134)	(0.00192)	(0.000799)	(0.000951)	(0.00137)
age_0047	0.114***	0.115***	0.112***	0.111***	0.110***	0.111***
	(0.00112)	(0.00135)	(0.00196)	(0.000814)	(0.000964)	(0.00139)
age_0048	0.128***	0.129***	0.129***	0.127***	0.126***	0.131***
	(0.00114)	(0.00136)	(0.00198)	(0.000826)	(0.000963)	(0.00142)
age_0049	0.141***	0.141***	0.140***	0.140***	0.140***	0.145***
	(0.00116)	(0.00139)	(0.00201)	(0.000839)	(0.000991)	(0.00143)
age_0050	0.152***	0.156***	0.156***	0.157***	0.154***	0.165***
	(0.00118)	(0.00141)	(0.00203)	(0.000863)	(0.00102)	(0.00144)
age_0051	0.166***	0.172***	0.172***	0.173***	0.168***	0.183***
	(0.00121)	(0.00143)	(0.00211)	(0.000871)	(0.00103)	(0.00148)
age_0052	0.179***	0.181***	0.185***	0.189***	0.186***	0.199***
	(0.00122)	(0.00146)	(0.00215)	(0.000888)	(0.00104)	(0.00152)
age_0053	0.189***	0.192***	0.194***	0.203***	0.198***	0.213***
	(0.00126)	(0.00148)	(0.00222)	(0.000919)	(0.00108)	(0.00160)
age_0054	0.201***	0.206***	0.210***	0.219***	0.214***	0.232***
	(0.00131)	(0.00154)	(0.00228)	(0.000957)	(0.00112)	(0.00166)
age_0055	0.215***	0.222***	0.231***	0.236***	0.230***	0.247***
	(0.00141)	(0.00167)	(0.00245)	(0.00103)	(0.00122)	(0.00177)
per_2005	− 0.0242***	− 0.0260***	− 0.0238***	− 0.0192***	− 0.0170***	− 0.0240***
	(0.000642)	(0.000762)	(0.00114)	(0.000504)	(0.000587)	(0.000871)
per_2006	− 0.0169***	− 0.0171***	− 0.0144***	− 0.00610***	− 0.00568***	− 0.00661***
	(0.000635)	(0.000760)	(0.00112)	(0.000488)	(0.000573)	(0.000830)
per_2007	− 0.000216	0.000413	− 0.00291***	0.00799***	0.00893***	0.00867***
	(0.000636)	(0.000758)	(0.00110)	(0.000515)	(0.000613)	(0.000861)
per_2008	0.0131***	0.0135***	0.00999***	0.0196***	0.0210***	0.0182***
	(0.000645)	(0.000774)	(0.00110)	(0.000480)	(0.000571)	(0.000798)
per_2009	0.0382***	0.0381***	0.0382***	0.0296***	0.0299***	0.0338***
	(0.000678)	(0.000826)	(0.00114)	(0.000499)	(0.000596)	(0.000810)
per_2010	0.0231***	0.0219***	0.0267***	0.0181***	0.0144***	0.0218***
	(0.000693)	(0.000850)	(0.00116)	(0.000506)	(0.000619)	(0.000821)
per_2011	0.00985***	0.00870***	0.0121***	− 0.000202	− 0.00292***	0.00448***
	(0.000709)	(0.000871)	(0.00118)	(0.000513)	(0.000625)	(0.000822)
per_2012	− 0.0150***	− 0.0115***	− 0.0146***	− 0.0250***	− 0.0256***	− 0.0249***
	(0.000737)	(0.000913)	(0.00120)	(0.000532)	(0.000657)	(0.000848)
per_2013	− 0.0193***	− 0.0183***	− 0.0234***	− 0.0318***	− 0.0326***	− 0.0338***
	(0.000785)	(0.000974)	(0.00128)	(0.000564)	(0.000702)	(0.000885)
per_2014	− 0.00104	0.00221**	− 0.00311**	− 0.00836***	− 0.00740***	− 0.0114***
	(0.000776)	(0.000964)	(0.00128)	(0.000558)	(0.000684)	(0.000894)
per_2015	− 0.000966	− 0.000717	− 0.000353	− 0.00220***	− 0.00314***	− 0.00375***
	(0.000757)	(0.000942)	(0.00125)	(0.000540)	(0.000667)	(0.000860)
per_2016	0.00136*	0.000306	0.00530***	0.00270***	0.00100	0.00390***
	(0.000734)	(0.000914)	(0.00121)	(0.000526)	(0.000648)	(0.000841)
per_2017	− 0.00782***	− 0.0118***	− 0.00761***	− 0.00387***	− 0.00404***	− 0.00443***
	(0.000710)	(0.000874)	(0.00118)	(0.000512)	(0.000625)	(0.000824)
per_2018	− 0.0113***	− 0.0120***	− 0.0136***	− 0.00435***	− 0.00223***	− 0.00597***
	(0.000682)	(0.000840)	(0.00114)	(0.000498)	(0.000607)	(0.000806)
per_2019	0.0111***	0.0121***	0.0116***	0.0231***	0.0253***	0.0240***
	(0.000656)	(0.000813)	(0.00109)	(0.000475)	(0.000586)	(0.000759)
_Ispanish_n_1				0.0196***	0.0417***	0.00220
				(0.000890)	(0.00113)	(0.00142)
_Iabroad_1				− 0.0741***	− 0.0827***	− 0.0683***
				(0.000735)	(0.000946)	(0.00113)
_Ivocationa_1				0.0812***	0.0775***	0.0887***
				(0.000382)	(0.000460)	(0.000631)
_Ipostcompu_1				0.0580***	0.0494***	0.0769***
				(0.000538)	(0.000684)	(0.000831)
_Iuniversit_1				0.111***	0.100***	0.143***
				(0.000533)	(0.000710)	(0.000789)
_Iccaa_resi_2				0.0420***	0.0664***	0.0241***
				(0.000902)	(0.00107)	(0.00146)
_Iccaa_resi_3				0.0169***	0.0582***	− 0.0353***
				(0.00105)	(0.00125)	(0.00168)
_Iccaa_resi_4				0.0740***	0.0738***	0.100***
				(0.000922)	(0.00117)	(0.00140)
_Iccaa_resi_5				− 0.0625***	− 0.0734***	− 0.0301***
				(0.000781)	(0.000986)	(0.00122)
_Iccaa_resi_6				0.0263***	0.0413***	0.00392*
				(0.00135)	(0.00166)	(0.00209)
_Iccaa_resi_7				− 0.000504	0.00282***	0.00350**
				(0.000799)	(0.000933)	(0.00141)
_Iccaa_resi_8				0.00157**	0.0111***	− 0.00205*
				(0.000714)	(0.000856)	(0.00119)
_Iccaa_resi_9				0.0968***	0.118***	0.0933***
				(0.000529)	(0.000631)	(0.000886)
_Iccaa_resi_10				− 0.0801***	− 0.0911***	− 0.0601***
				(0.00116)	(0.00137)	(0.00203)
_Iccaa_resi_11				− 0.0600***	− 0.0391***	− 0.0614***
				(0.000695)	(0.000839)	(0.00113)
_Iccaa_resi_12				0.0417***	0.0478***	0.0457***
				(0.000540)	(0.000662)	(0.000885)
_Iccaa_resi_13				− 0.0170***	− 0.0132***	− 0.0144***
				(0.000954)	(0.00111)	(0.00169)
_Iccaa_resi_14				0.151***	0.173***	0.130***
				(0.00112)	(0.00134)	(0.00185)
_Iccaa_resi_15				0.175***	0.182***	0.172***
				(0.000719)	(0.000861)	(0.00120)
_Iccaa_resi_16				0.0505***	0.0633***	0.0559***
				(0.00160)	(0.00194)	(0.00259)
_Iccaa_resi_17				− 0.00102*	0.00337***	0.00530***
				(0.000602)	(0.000727)	(0.000999)
_Iccaa_resi_18				0.0886***	0.0695***	0.123***
				(0.00333)	(0.00402)	(0.00560)
_Itemporary_1				− 0.0859***	− 0.104***	− 0.0643***
				(0.000373)	(0.000467)	(0.000599)
exp				− 0.00227***	− 0.00333***	− 0.00280***
				(2.67e-05)	(3.35e-05)	(4.19e-05)
_Iagricultu_1				− 0.146***	− 0.162***	− 0.132***
				(0.00182)	(0.00194)	(0.00481)
_Iconstruct_1				− 0.00109**	− 0.0169***	− 0.0225***
				(0.000516)	(0.000552)	(0.00156)
_Itrade_1				− 0.159***	− 0.129***	− 0.152***
				(0.000480)	(0.000566)	(0.000837)
_Itourism_1				− 0.181***	− 0.185***	− 0.0741***
				(0.000601)	(0.000808)	(0.000978)
_Itransp_1				− 0.0619***	− 0.0782***	− 0.0301***
				(0.000658)	(0.000713)	(0.00151)
_Ihealth_1				− 0.212***	− 0.179***	− 0.149***
				(0.000637)	(0.00113)	(0.000885)
_Ieducat_1				− 0.282***	− 0.266***	− 0.219***
				(0.000980)	(0.00166)	(0.00128)
_Ifinancial_1				0.135***	0.104***	0.212***
				(0.000702)	(0.000863)	(0.00117)
_Is_company_1				− 0.161***	− 0.159***	− 0.115***
				(0.000531)	(0.000670)	(0.000886)
_Io_service_1				− 0.148***	− 0.118***	− 0.134***
				(0.000680)	(0.000831)	(0.00109)
_Imedium_ma_				0.104***	0.0960***	0.0879***
				(0.000604)	(0.000710)	(0.00101)
_Ihigh_manu_1				0.191***	0.159***	0.147***
				(0.000534)	(0.000614)	(0.00107)
_Ilow_nonma_1				0.0801***	0.0603***	0.151***
				(0.000617)	(0.000877)	(0.000914)
_Imedium_no_1				0.335***	0.336***	0.358***
				(0.000598)	(0.000747)	(0.000943)
_Ihigh_nonm_1				0.562***	0.513***	0.619***
				(0.000695)	(0.000891)	(0.00109)
_Ib10_19_1				0.0760***	0.0671***	0.0777***
				(0.000515)	(0.000605)	(0.000903)
_Ib20_49_1				0.121***	0.117***	0.117***
				(0.000473)	(0.000560)	(0.000812)
_Ib50_249_1				0.189***	0.194***	0.173***
				(0.000443)	(0.000541)	(0.000731)
_Ib250_499_1				0.246***	0.254***	0.233***
				(0.000596)	(0.000742)	(0.000933)
_Imore500_1				0.286***	0.292***	0.272***
				(0.000501)	(0.000642)	(0.000772)
Observations	4,391,680	2,738,896	1,652,314	4,230,395	2,628,345	1,602,038

This table displays the trended age-period-cohort effects using as dependent variable the logarithm of monthly earnings. Robust standard errors in parentheses ****p* < 0.01, ***p* < 0.05, **p* < 0.1.

## Footnotes

1 Note that the term inequality used throughout this paper is referred to those differences arising between cohorts.

2 The term economic crisis used in this article refers to the economic and financial crisis that took place during the period 2008–2013.

3 This model is used by Vera-Toscano and Meroni (2021) to analyse overeducation and skill mismatch.

4 In Appendix, Table 1 shows trends in earnings by period and age in our whole sample. It follows the progression of cohorts’ down the diagonal. However, this progression of cohorts incorporates both age and period effects. Our modeling strategy allows us to move beyond the analytical limitation of this table to analyze age, period, and cohort effects net of the other two factors.

5 Data on part-time work is insufficient to calculate hourly wages.

6 We will use the terms earnings and income interchangeably in the analysis.

7 We also estimate the APCD model using annual earnings as dependent variable and we find similar results.

8 Mainly promoted by the General Education Law of 1970, covering the entire national educational system, that is, the different educational levels.
